# Loss and Gain of Aqp10 Paralogs With Broad Solute Selectivity in Anguillid Eels

**DOI:** 10.1093/gbe/evaf169

**Published:** 2025-09-12

**Authors:** Ayumi Nagashima, Shinichiro Hidaka, Chihiro Ota, Daisuke Yamanaka, Koichi Ito, Tsutomu Nakada, Tadaomi Furuta, Akira Kato

**Affiliations:** School of Life Science and Technology, Institute of Science Tokyo, Yokohama, Kanagawa 226-8501, Japan; School of Life Science and Technology, Tokyo Institute of Technology, Yokohama, Kanagawa 226-8501, Japan; School of Life Science and Technology, Institute of Science Tokyo, Yokohama, Kanagawa 226-8501, Japan; School of Life Science and Technology, Tokyo Institute of Technology, Yokohama, Kanagawa 226-8501, Japan; School of Life Science and Technology, Institute of Science Tokyo, Yokohama, Kanagawa 226-8501, Japan; School of Life Science and Technology, Tokyo Institute of Technology, Yokohama, Kanagawa 226-8501, Japan; Department of Food and Physiological Models, Graduate School of Agricultural and Life Sciences, The University of Tokyo, Kasama, Ibaraki 319-0206, Japan; Y's Data Science Co., Ltd., Tokyo 113-0033, Japan; Department of Food and Physiological Models, Graduate School of Agricultural and Life Sciences, The University of Tokyo, Kasama, Ibaraki 319-0206, Japan; Division of Instrumental Analysis, Research Center for Advanced Science and Technology, Shinshu University, Matsumoto, Nagano 390-8621, Japan; School of Life Science and Technology, Institute of Science Tokyo, Yokohama, Kanagawa 226-8501, Japan; School of Life Science and Technology, Tokyo Institute of Technology, Yokohama, Kanagawa 226-8501, Japan; School of Life Science and Technology, Institute of Science Tokyo, Yokohama, Kanagawa 226-8501, Japan; School of Life Science and Technology, Tokyo Institute of Technology, Yokohama, Kanagawa 226-8501, Japan

**Keywords:** aquaporin 10, European eel, tandem duplication, solute selectivity, neo-subfunctionalization

## Abstract

Aquaporin (Aqp) 10 is a member of the aquaglyceriporin family, which transports small, uncharged solutes in addition to water. Although the solute selectivity of aquaglyceroporins varies, the mechanism of solute selectivity has not yet been fully elucidated. The common ancestor of ray-finned fish possessed two paralogous genes for aquaporin 10, *aqp10.1* and *aqp10.2*, which produce Aqps with different solute selectivities. Most teleosts possess one or more ohnologs derived from *aqp10.1* and *aqp10.2*; however, the common ancestor of Anguilliformes species lost all *aqp10.1*-derived ohnologs. Anguilliformes species, except *Anguilla* species, have one *aqp10.2b*, but recent tandem duplications in the European eel have generated three *aqp10.2b* paralogs (*aqp10.2b1–aqp10.2b3*), whose activities remain ambiguous. In this study, we found that the four sites forming the aromatic/arginine (ar/R) selectivity filter in European eel Aqp10.2b1 were identical to those in Aqp10.2b of other species. However, the Y residue at position 3 was replaced with G in the ar/R selectivity filter of Aqp10.2b2 and b3. When expressed in *Xenopus* oocytes, Aqp10.2b2 and b3 showed higher permeability to urea and boric acid than Aqp10.2b1, indicating that Aqp10.2b2 and b3 acquired broad solute selectivity similar to that of Aqp10.1, which was lost in the ancestral Anguilliformes species. Urea and boric acid permeabilities of Aqp10.2b1 increased when the Y residue at position 3 of the ar/R selectivity filter was replaced with G. Overall, our results outline the history of the loss and gain of Aqp10 paralogs with broad solute selectivity in anguillid eels.

Significance During evolution, vertebrate gene families have undergone various duplications and losses, known as birth-and-death evolution. Vertebrate aquaporin gene (*aqp*) family has undergone several lineages- and species-specific duplications and losses; however, their roles have not been fully understood. In this study, we evaluated the deletion of an *aqp10* paralog specifically observed in Anguilliformes species and a lineage-specific tandem duplication of another *aqp10* paralog in *Anguilla*. We measured the activity of the protein encoded by each *aqp10* paralog and identified a process by which *Anguilla* species reacquired the gene encoding Aqp10 with solute selectivity similar to that of the Aqp lost in ancestral Anguilliformes species. This study provides insights into the gain, loss, and sub-neofunctionalization of *aqp* genes in *Anguilla* species approximately 16 million years ago, which is the estimated time of divergence of *aqp10.2b1* and *aqp10.2b2*/*b3*.

## Introduction

Aquaporins (Aqps) are a family of water channels that involve water-specific aquaporins that are only permeable to water and aquaglyceroporins that are permeable to water and uncharged low-molecular-weight substances, such as glycerol (Agre et al. 2002; Login and Nejsum 2023). In mammals, Aqp-3, -7, -9, and -10 are aquaglyceroporins that have been shown to contribute to glycerol handling and urea metabolism in the skin, intestines, kidneys, liver, and adipose tissue (Agre et al. 2002; Login and Nejsum 2023). (Note that, in this article, protein name abbreviations of all species are shown with the first letter capitalized, and gene names of all species are shown as lowercase and italicized.) Aqp10 is one of the members of the aquaglyceroporin subfamily of water channels, and is widely distributed in vertebrates (Finn et al. 2014; Finn and Cerda 2015; Yilmaz et al. 2020). Aqp10 plays an important role in glycerol metabolism and is expressed in the human intestine and adipose tissues (Mobasheri et al. 2004; Gotfryd et al. 2018). Aqp10 paralogs exhibit diversity in the number of genes and solute selectivity among different species. Lobe-finned fish and most tetrapods possess a single *aqp10* gene, whereas mice, cattle, and whales lack or pseudogenize *aqp10* (Morinaga et al. 2002; Tanaka et al. 2015; Nagashima et al. 2025a). Owing to the absence of *aqp10* in mice, prior research on *aqp10* analysis using knockout mice has not been conducted. Yilmaz et al. (2020) showed that ray-finned fish tandemly duplicated *aqp10* in their ancestral species and acquired *aqp10.1* and *aqp10.2*. Polypterus and gar, known as basal ray-finned fishes, retain *aqp10.1* and *aqp10.2* in tandem. Therefore, the ancestral teleost species presumably acquired two ohnologs each and four *aqp10* paralogs (*aqp10.1a* and *aqp10.1b* from *aqp10.1* and *aqp10.2a* and *aqp10.2b* from *aqp10.2*) (Yilmaz et al. 2020) via teleost-specific whole-genome duplication (Glasauer and Neuhauss 2014; Ravi and Venkatesh 2018). In previous studies, names for *aqp10.1*, *aqp10.1a*, and *aqp10.1b* were *aqp10a* (or *aqp10aa*), *aqp10aa*, and *aqp10ba*, respectively (Tingaud-Sequeira et al. 2010; Yilmaz et al. 2020). *aqp10.2*, *aqp10.2a*, and *aqp10.2b* were previously known as *aqp10b* (or *aqp10ab*), *aqp10ab*, and *aqp10bb*, respectively. In this study, the gene symbols of tandem duplicates are appended with “.1” or “.2,” whereas those of ohnologs—gene duplicates originating from whole-genome duplication—are indicated by “a” or “b,” following the Zebrafish Nomenclature Conventions (http://zfin.org/) (Imaizumi et al. 2024; Nagashima et al. 2025b). Subsequent gene deletions left 2 to 3 *aqp10* paralogs in teleost species (Yilmaz et al. 2020). Many teleosts, such as zebrafish, tilapia, medaka, stickleback, and pufferfish, possess *aqp10.1a* and *aqp10.2b* on separate chromosomes. Cod and electric eel possess two *aqp10.1* ohnologs and one *aqp10.2* ohnolog. Herring and shad possess one *aqp10.1* ohnolog and two *aqp10.2* ohnologs. Analysis of the activity of the *aqp10* gene products has shown that Sarcopterygii Aqp10s and Aqp10.1-derived Aqps in ray-finned fish exhibit broad solute selectivity, as they are permeable to water, glycerol, urea, and boric acid, whereas Aqp10.2-derived Aqps in ray-finned fish exhibit narrow solute selectivity, as they are only permeable to water and glycerol, but not or weakly permeable to urea and boric acid (Imaizumi et al. 2024). These results indicate that water, glycerol, urea, and boric acid permeabilities are plesiomorphic activities of Aqp10s and that most ray-finned fish-specific Aqp10.2 paralogs have secondarily reduced or lost urea and boric acid permeability.

The mechanisms that control the permeability of both water and solutes through aquaporins have been clarified through structural biological analyses such as X-ray crystallography, molecular dynamics, and cryo-electron microscopy (cryo-EM) (Hub and de Groot 2008; Eriksson et al. 2013; Kreida and Törnroth-Horsefield 2015; Gotfryd et al. 2018; Kozai et al. 2025). Some aquaporins can form homotetramers in the membrane with one channel pore in each subunit. Each subunit forms a narrow pore with six transmembrane domains. The narrow pore region is formed from four amino acid residues containing aromatic and arginine residues and is called an aromatic/arginine (ar/R) selectivity filter. The pore size plays an important role in controlling the permeability of water and other molecules and determines the differences between water-selective aquaporins and aquaglyceroporins, whereas the amino acid residues in the ar/R selectivity filter play an important role in determining the specificity between water-specific aquaporins and aquaglyceroporins. There are two types of aquaglyceroporins: those with relatively high solute selectivity that are highly permeable to glycerol, and those with low solute selectivity that are highly permeable to glycerol, urea, and boric acid. Aqp3 is highly permeable to glycerol and exhibits some urea permeability; however, Aqp7, Aqp9, and Aqp10 are highly permeable to urea, boric acid, and glycerol (Kitchen et al. 2019; Ushio et al. 2022; Kumagai et al. 2023; Nagashima et al. 2025b). Although Aqp8 is not typically classified as an aquaglyceroporin owing to its inability to transport glycerol, some fish Aqp8 channels have been shown to permeate glycerol (Engelund et al. 2013). Furthermore, fish Aqp8 channels are permeable to urea (Tingaud-Sequeira et al. 2010; Engelund et al. 2013; Kumagai et al. 2023). Thus, the solute selectivity of aquaporins is diverse; however, the underlying molecular mechanisms are not fully understood.

Recent studies have shown that the size of the amino acid residues in the ar/R selectivity filter and pore width are involved in the solute selectivity of aquaglyceroporins (Hub and de Groot 2008), and it has become clear that this selectivity can be predicted by the primary sequence (Hub and de Groot 2008; Kitchen et al. 2019; Nagashima et al. 2025b). Human Aqp3 is highly permeable to glycerol but not urea, and the four amino acid residues of the ar/R selectivity filter are F, G, Y, and R. The Y to A mutant at position 3 of the ar/R selectivity filter of human Aqp3 shows good permeability to urea and glycerol (Kitchen et al. 2019). In regard to Aqp10 in bony vertebrates, position 2 of the ar/R selectivity filter of Aqp10 is conserved as a small amino acid residue, and position 4 is conserved as an R (Nagashima et al. 2025b). However, there was diversity between positions 1 and 3. *Xenopus* Aqp10 and ray-finned fish Aqp10.1 exhibit high permeability to glycerol and urea, showing broad solute selectivity. Positions 1 and 3 of the ar/R selectivity filter of *Xenopus* Aqp10 are G and Y, respectively, and those of ray-finned fish Aqp10.1 are F and A, respectively. On the other hand, positions 1 and 3 of the ar/R selectivity filter of the ray-finned fish Aqp10.2, which selectively permeates glycerol and exhibits narrow solute selectivity, are F and Y, respectively, and the mutants in which one of these two positions is replaced with G acquire high urea permeability and broad solute selectivity (Nagashima et al. 2025b). These results show that the presence of bulky amino acid residues at positions 1 and 3 of the ar/R selectivity filter determines the narrow solute selectivity and reduces permeability to urea and boric acid. Furthermore, a known correlation exists between the Aqp permeability to urea and boric acid: Aqps with high urea permeability also exhibit high boric acid permeability, while those with low urea permeability show low boric acid permeability. Nevertheless, a certain degree of independence exists between the permeability of Aqp to glycerol and urea or boric acid (Kumagai et al. 2023). At the same time, it has been suggested that the breadth of solute selectivity of Aqp10 can be predicted by examining the amino acid residues at positions 1 and 3 of the ar/R selectivity filter encoded in the genomic databases of a large number of bony vertebrate species (Nagashima et al. 2025b).

Among ray-finned fish, Yilmaz et al. (2020) showed that Anguilliformes are exceptionally deficient in *aqp10.1*-derived genes. The giant moray, Kaup's arrowtooth eel, and European conger only have *aqp10.2b* as an *aqp10* ortholog. Yilmaz et al. also found that *Anguilla* species, such as the European eel (*Anguilla anguilla*), Japanese eel (*Anguilla japonica*), and American eel (*Anguilla rostrata*), acquired multiple *aqp10* genes via tandem duplication of *aqp10.2b*. Therefore, ancestral Anguilliformes lost all *aqp10* paralogs, other than *aqp10.2b*, and the ancestral *Anguilla* species acquired the lineage-specific multiple *aqp10.2b* paralogs. However, to date, no studies have examined the differences in the functions of the Aqp10.2b paralogs in *Anguilla* species.

In a previous study, we analyzed 293 amino acid sequences of Aqp10s in the genome databases of various bony vertebrates, classified them as Sarcopterygii Aqp10, Actinopterygii Aqp10.1, and Actinopterygii Aqp10.2, and extracted the amino acid residues at positions 1 and 3 in the ar/R selectivity filter (Nagashima et al. 2025b). Almost all Sarcopterygii Aqp10s and Actinopterygii Aqp10.1s had one or no aromatic residues at positions 1 and 3 in the ar/R selectivity filter. In contrast, most Actinopterygii Aqp10.2s had two aromatic residues at positions 1 and 3 in the ar/R selectivity filter. A few exceptions to this analysis include the European eel Aqp10.2b2 and b3. As described by Yilmaz et al. (2020), these *Anguilla*-specific paralogs are generated via tandem duplications in the ancestral *Anguilla* species. However, European eel Aqp10.2b2 and b3 have F and G residues at positions 1 and 3, respectively, in the ar/R selectivity filter. In contrast, European eel Aqp10.2b1 had F and Y residues at positions 1 and 3, respectively, in the ar/R selectivity filter, as observed in most other Actinopterygii Aqp10.2s. This result suggests that the solute selectivities of European eel Aqp10.2b2 and b3 are different from that of Aqp10.2b1 and that the European eel acquired Aqp10 paralogs with different solute selectivities via tandem gene duplication. To verify this hypothesis, we analyzed the activity of European eel Aqp10.2b paralogs expressed in *Xenopus* oocytes and found that Aqp10.2b2 and b3 acquired high urea and boric acid permeabilities by substituting an aromatic amino acid residue for a glycine residue in the ar/R selectivity filter.

## Results

### *aqp10* Genes in Anguilliformes Species

**Fig. 1.**
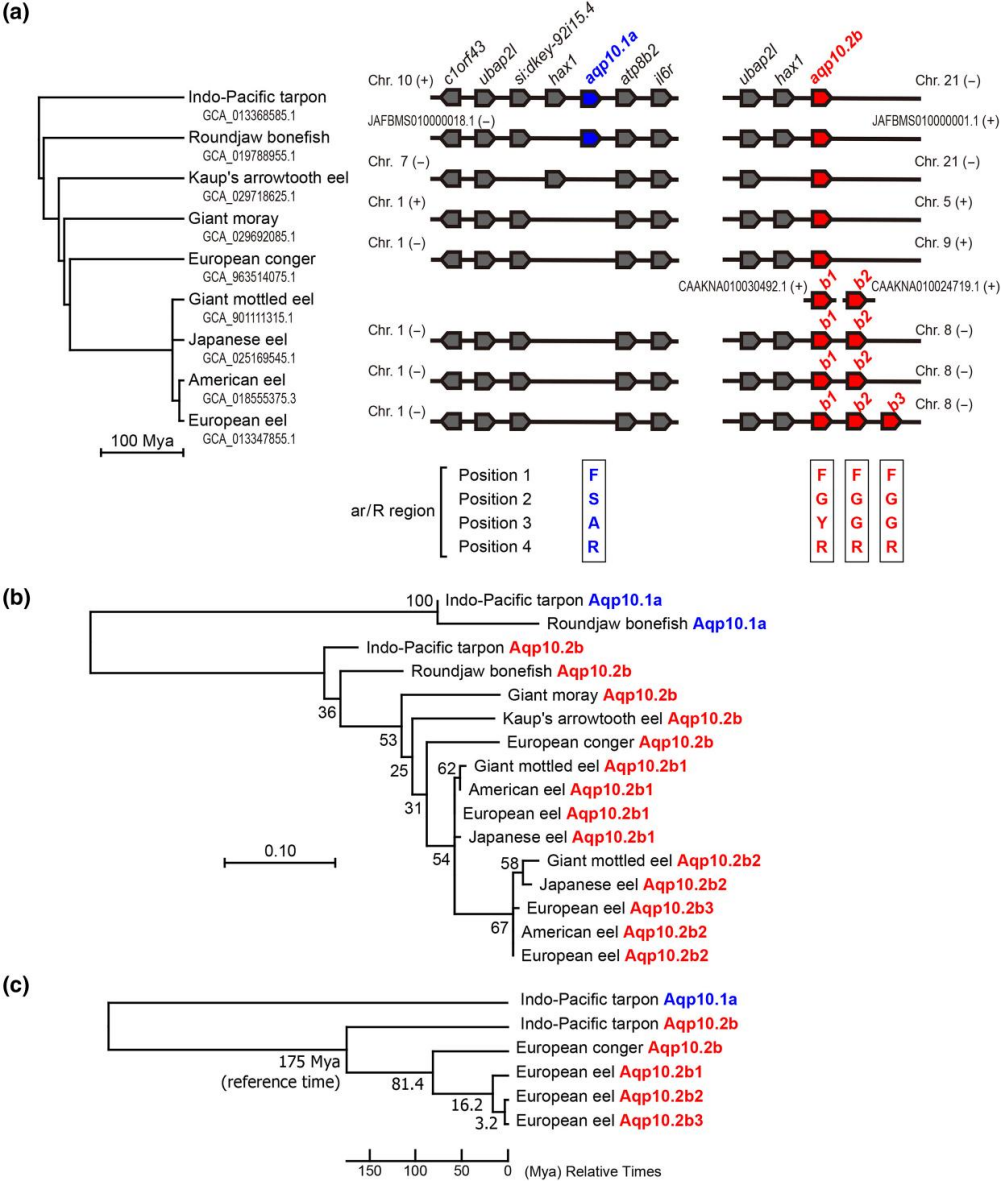
Synteny and phylogenetic analyses of *aqp10.2b* paralogs in European eel. a) Synteny analyses of *aqp10* in Indo-Pacific tarpon, roundjaw bonefish, and Anguilliformes. (+) and (−) (right and left orientations, respectively) genome sequences of various species in the National Center for Biotechnology Information (NCBI) and Ensembl databases are illustrated by horizontal lines. Arrow-shaped boxes indicate the orientation of each gene. Phylogeny of bony vertebrate species based on the TimeTree database (http://www.timetree.org/) (Kumar et al. 2017) is shown on the left. The four amino acid residues in the aromatic/arginine (ar/R) selectivity filter in each Aqp10 are shown below the synteny data. Accession numbers of genes are listed in Table S1. b) Phylogenetic analyses of Aqp10 amino acid sequences in Elopomorpha species. The amino acid sequences were aligned using ClustalW software, and a phylogenetic tree was constructed using the maximum-likelihood method with MEGA software. Numbers indicate the bootstrap values, and scale bar represents the genetic distance of amino acid substitutions per site. Accession numbers of proteins are listed in Table 1. c) Molecular clock analysis of *aqp10.2b* paralogs in European eel. Divergence time between Elopiformes (Indo-Pacific tarpon) and Anguilliformes (European conger and European eel) species 175 Mya was used as a reference. Numbers indicate the estimated time points in Mya.

Composition of *aqp10* in Anguilliformes has been previously reported (Yilmaz et al. 2020). Here, we confirmed the results using synteny and phylogenetic analyses. Synteny analysis results of Anguilliformes (Kaup's arrowtooth eel [*Synaphobranchus kaupii*], giant moray [*Gymnothorax javanicus*], European conger [*Conger conger*], giant mottled eel [*Anguilla marmorata*], Japanese eel [*A. japonica*], American eel [*A. rostrata*], and European eel [*A. anguilla*]) and their related species, such as Roundjaw bonefish (*Albula glossodonta* in Albuliformes) and Indo-Pacific tarpon (*Megalops cyprinoides* in Elopiformes), are shown in Fig. 1a and Table S1. Similar to many other teleost species, roundjaw bonefish and Indo-Pacific tarpon had one *aqp10.1a* and one *aqp10.2b* at separate chromosomal loci (Fig. 1a). As reported by Yilmaz et al., *aqp10.1a* was not found at the same locus in Anguilliformes species (Fig. 1a). One *aqp10.2b* was present in Kaup's arrowtooth eel, giant moray, and European conger, whereas three paralogs for *aqp10.2b*, *aqp10.2b1*, *aqp10.2b2*, and *aqp10.2b3*, were tandemly present in the European eel (Fig. 1a). In Japanese and American eels, two paralogs, *aqp10.2b*, *aqp10.2b1*, and *aqp10.2b2*, were found at the same locus. Due to the short assembly, synteny analysis of the giant mottled eel could not be performed in this study. However, a BLAST search confirmed the presence of two paralogs, *aqp10.2b1* and *aqp10.2b2*, in giant mottled eel. The accession numbers for the amino acid sequences derived from the predicted protein-coding regions are listed in Table 1.

**Table 1:** Aquaglyceroporins (Aqp10s) analyzed/identified in this study

Species	Protein name in this study	Accession no.	Other name in past studies
Indo-pacific tarpon (*Megalops cyprinoides*)	Aqp10.1a	XM_036538676.1	Aqp10aa (Yilmaz et al. 2020)
	Aqp10.2b	XM_036516187.1	Aqp10bb (Yilmaz et al. 2020)
Roundjaw bonefish (*Albula glossodonta*)	Aqp10.1a	BR002456	Aqp10aa (Yilmaz et al. 2020)
	Aqp10.2b	BR002457	Aqp10bb (Yilmaz et al. 2020)
Kaup's arrowtooth eel (*Synaphobranchus kaupii*)	Aqp10.2b	KAJ8335135.1 (amino-acid residues 281 to 599)	Aqp10bb (Yilmaz et al. 2020)
Giant moray (*Gymnothorax javanicus*)	Aqp10.2b	KAJ8273543.1	Aqp10bb (Yilmaz et al. 2020)
European conger (*Conger conger*)	Aqp10.2b	XM_061254624.1	Aqp10bb (Yilmaz et al. 2020)
Giant mottled eel (*Anguilla marmorata*)	Aqp10.2b1	BR002453	Aqp10bb1 (Yilmaz et al. 2020)
	Aqp10.2b2	BR002454 (5′ end incomplete)	Aqp10bb2 (Yilmaz et al. 2020)
Japanese eel (*Anguilla japonica*)	Aqp10.2b1	AB378503.1	Aqp10 (Kim et al. 2010) Aqp10bb1 (Yilmaz et al. 2020)
	Aqp10.2b2	BR002452	Aqp10bb2 (Yilmaz et al. 2020)
American eel (*Anguilla rostrata*)	Aqp10.2b1	XM_064348527.1	Aqp10bb1 (Yilmaz et al. 2020)
	Aqp10.2b2	XM_064348525.1	Aqp10bb2 (Yilmaz et al. 2020)
European eel (*Anguilla anguilla*)	Aqp10.2b1	XM_035428387.1	AQPe (AJ784153) (Martinez et al. 2005b) Aqp10bb1 (Yilmaz et al. 2020)
	Aqp10.2b2	XM_035430495.1	Aqp10bb2 (Yilmaz et al. 2020)
	Aqp10.2b3	XM_035430494.1	Aqp10bb3 (Yilmaz et al. 2020)

Each sequence is shown in Table S3.

Yilmaz et al. reported *aqp10bb1*, *aqp10bb2*, and *aqp10bb3* as paralogs of the *Anguilla aqp10*, we refer to them as *aqp10.2b1*, *aqp10.2b2*, and *aqp10.2b3*, respectively, as described in the Introduction. In this study, we have used *A. rostrata* EN2019 (GCA_018555375.3) genome data, registered in the National Center for Biotechnology Information (NCBI) Genome Data Viewer as the genome database for the American eel. This is one of the two genome datasets for the American eel and the other is *A. rostrata* LakeOntario-01-2011 (GCA_001606085.1). In the *A. rostrata* EN2019 (GCA_018555375.3) analyzed in this study, there were two *aqp10.2b* paralogs, *aqp10.2b1* and *aqp10.2b2*, that are tandemly located (Fig. 1a). In an analysis by Yilmaz et al. (2020), three *aqp10* paralogs of the American eel were reported and named *aqp10bb1*, *aqp10bb2*, and *aqp10bb3*; however, we were unable to find the three *aqp10* paralogs within the same genome database of *A. rostrata*. The sequences in the genome database *A. rostrata* LakeOntario-01-2011 (GCA_001606085.1) were too short to observe the two *aqp10.2b* paralogs within a single scaffold, but we were able to obtain a partial sequence corresponding to *aqp10.2b1* (*aqp10bb1*) and one sequence corresponding to *aqp10.2b2* (*aqp10bb2*) from the BLAST analysis of the genome data *A. rostrata* LakeOntario-01-2011 (GCA_001606085.1). At present, there are no data indicating the presence of three *aqp10.2b* paralogs within the same individual of *A. rostrata*. The nucleotide sequence of *aqp10.2b2* in *A. rostrata* LakeOntario-01-2011 (GCA_001606085.1) was 98% identical to that of *aqp10.2b2* in *A. rostrata* EN2019 (GCA_018555375.3) and 100% identical to the American eel *aqp10bb2* sequence reported by Yilmaz et al. (2020). A dot plot analysis of *aqp10.2b2* and *aqp10.2b3* from the European eel and *aqp10.2b2* from the two American eel genomes revealed that the European eel *aqp10.2b2* and the American eel *aqp10.2b2* (EN2019, GCA_018555375.3) are closely related, and the European eel *aqp10.2b3* and the American eel *apq10.2b2* (LakeOntario-01-2011, GCA_018555375.3) appear to be closely related (Fig. S1). It is unclear at this point whether the *A. rostrata* has *aqp10.2b2* with polymorphisms at the gene or allelic level, or if it has *aqp10.2b2* and *aqp10.2b3*; however, the presence of these genes has not been demonstrated in a single *A. rostrata* individual. Further analyses are required to resolve this issue. Yilmaz et al. (2020) also reported that *A. rostrata aqp10bb1* is a pseudogene; however, this conclusion is likely based on an analysis of a partial *aqp10.2b1* sequence present in the *A. rostrata* LakeOntario-01-2011 (GCA_001606085.1) genome data. The *A. rostrata* EN2019 (GCA_018555375.3) genome data contains full-length *aqp10.2b1*, thus suggesting that this gene is not a pseudogene.

### Altered Amino Acid Residues in the ar/R Selectivity Filters of Aqp10.2b2 and b3 in *Anguilla* Species

Amino acid sequences of Aqp10 s in Anguilliformes, Roundjaw bonefish, and Indo-Pacific tarpons were aligned, as shown in Fig. S2. Four sites forming the ar/R selectivity filter were predicted from this alignment (Fig. 1a). The amino acid residues at positions 1, 2, 3, and 4 in the ar/R selectivity filter were conserved as F, S, A, and R, respectively, in Aqp10.1a of the Indo-Pacific tarpon and Roundjaw bonefish (Fig. 1a). The same residues were conserved as F, G, Y, and R, respectively, in Aqp10.2b of the Indo-Pacific tarpon, Roundjaw bonefish, and Anguilliformes species, and Aqp10.2b1 of *Anguilla* species (Fig. 1a). In contrast, they were F, G, G, and R, respectively, in Aqp10.2b2 and b3 of *Anguilla* species. These results suggest that the Y-to-G substitution at position 3 in the ar/R selectivity filter has occurred in Aqp10.2b2 and b3 and that the substitution may alter the solute selectivity of these Aqp10s.

### Phylogenetic and Molecular Clock Analyses of the European Eel Aqp10.2b Paralogs

Next, a phylogenetic tree was constructed based on the amino acid sequences of Aqp10s in Anguilliformes species, Roundjaw bonefish, and Indo-Pacific tarpon (Fig. 1b). The Aqp10.2b paralogs in *Anguilla* species formed a single clade after separation from Aqp10.2b in the European conger, confirming that the tandem duplication of Aqp10.2b is specific to *Anguilla* species in Anguilliformes. Subsequently, we constructed another phylogenetic tree based on the nucleotide sequences of Aqp10s and performed molecular clock analysis to estimate the age of gene duplication using the 175 Mya divergence time between Anguilliformes and Indo-Pacific tarpon as a reference (Fig. 1c). The divergence time of *aqp10.2b1* and *aqp10.2b2*/*b3* was estimated as 16.2 Mya, whereas that of the European eel *aqp10.2b2* and *aqp10.2b3* was estimated as 3.2 Mya.

The relationships between *aqp10.2b1*, *aqp10.2b2*, and *aqp10.2b3* in *Anguilla* species were also confirmed by dot plot analysis (Fig. S3). As these genes were located in tandem at the same chromosomal locus (Fig. 1a), the corresponding regions were compared among European, Japanese, and American eels. The region encoding *aqp10.2b1* was well conserved among the three *Anguilla* species, thus confirming that the *aqp10.2b1* genes in these species are orthologs. Additionally, the regions encoding European eel *aqp10.2b2* and *aqp10.2b3* and Japanese and American eel *aqp10.2b2* were well conserved, confirming that these genes are closely related.

### Difference in Solute Permeabilities Between European Eel Aqp10.2b1, b2, and b3

**Fig. 2.**
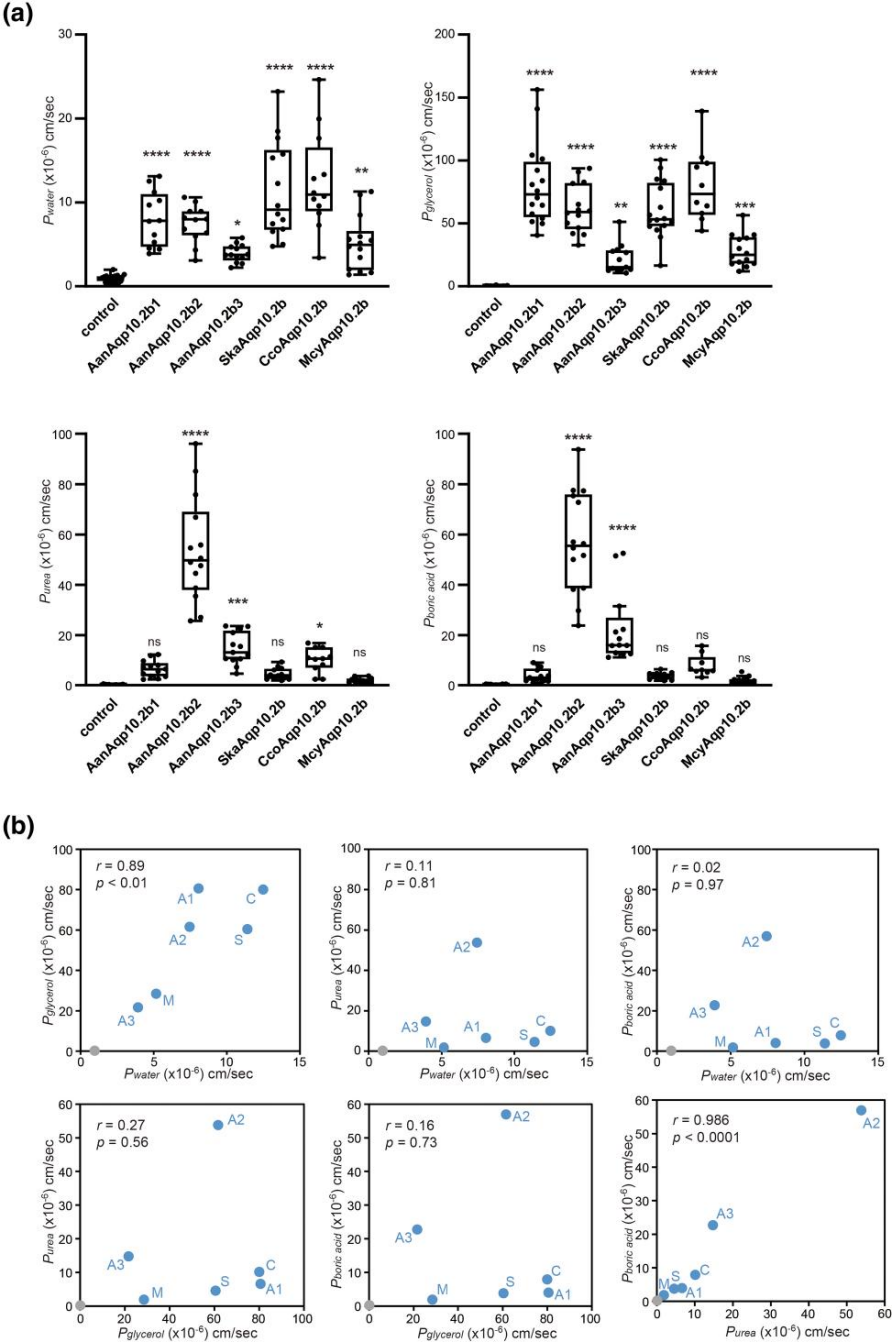
Activities of Aqp10.2b orthologs in European eel, Kaup's arrowtooth eel, European conger, and Indo-Pacific tarpon. a) Water and solute permeabilities of AanAqp10.2b1, AanAqp10.2b2, AanAqp10.2b3, SkaAqp10.2b, CcoAqp10.2b, and McyAqp10.2b. Water (*P*_water_), glycerol (*P*_glycerol_), urea (*P*_urea_), and boric acid (*P*_boric acid_) permeabilities of oocytes expressing Aqp10.2b orthologs compared to those of control oocytes. Data were plotted using box-and-whisker plots (median: 25% to 75%ile; 1.5× interquartile). Statistical significance was assessed via one-way analysis of variance (ANOVA), followed by the Holm–Sidak post-hoc test (****P < 0.0001, ***P < 0.001, **P < 0.01, and *P < 0.05). All samples are listed in Tables S4 and S6. b) Scatter plots and Pearson's correlation coefficient (*r*) values for water, glycerol, urea, and boric acid permeabilities of oocytes expressing Aqp10.2b orthologs. The average water and solute permeabilities (*P*_water_, *P*_glycerol_, *P*_urea_, and *P*_boric acid_; Table S6) of oocytes expressing Aqp10.2bs are plotted in blue, whereas those of water-injected oocytes are plotted in gray. The correlations (*r*) calculated from the average values of Aqp10.2bs and control (*n* = 17 to 20) are shown with the *P* values in black. Aan, *Anguilla anguilla*; Ska, *Synaphobranchus kaupii*; Cco, *Conger conger*; Mcy, *Megalops cyprinoides*; A1, AanAqp10.2b1; A2, AanAqp10.2b2; A3, AanAqp10.2b3; S, SkaAqp10.2b; C, CcoAqp10.2b; M, McyAqp10.2b.

In order to confirm whether the differences in the ar/R selectivity filter between *Anguilla* Aqp10.2b paralogs caused differences in solute selectivity, three European eel Aqp10.2b paralogs (Aqp10.2b1, b2, and b3) were expressed in *Xenopus* oocytes, and their activity was then analyzed using a swelling assay. First, we analyzed the water permeability of oocytes expressing European eel Aqp10.2b1, b2, or b3. Oocytes expressing Aqp10.2b paralogs showed significant volume gain and increase in *P_water_* in hypoosmotic solution (Fig. 2), confirming that these Aqp10s act as water channels in the plasma membranes of oocytes.

Glycerol, urea, and boric acid permeabilities of oocytes expressing Aqp10.2b paralogs were similarly analyzed using a swelling assay with an iso-osmotic solution containing the solutes. In an iso-osmotic solution containing glycerol, all oocytes expressing Aqp10.2b paralogs showed significant volume gain and increased *P_glycerol_* (Fig. 2), indicating that these Aqp10 s are permeable to glycerol. In an iso-osmotic solution containing urea and boric acid, Aqp10.2b2 and b3, but not b1, showed significant volume gain and increased *P_urea_* and *P_boric acid_* (Fig. 2), indicating that Aqp10.2b2 and b3, but not b1, are permeable to urea and boric acid.

**Fig. 3.**
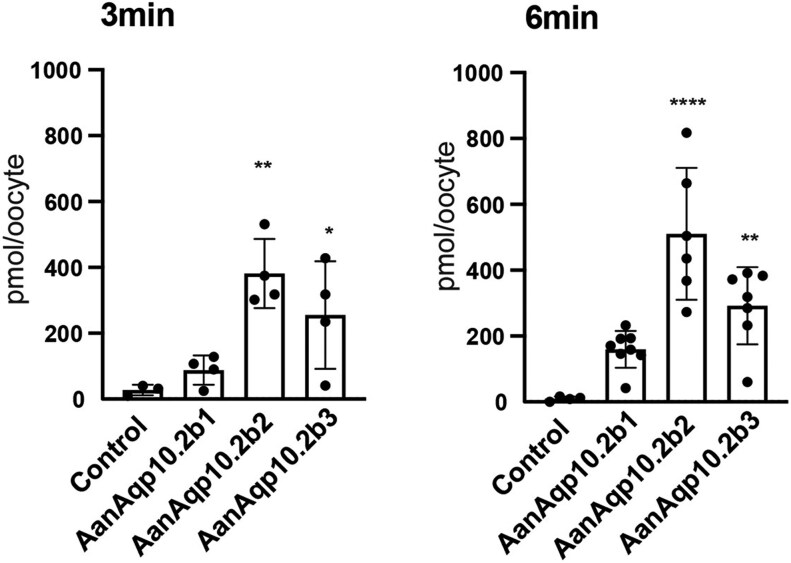
Uptake assays for ^15^N-labeled urea by oocytes expressing European eel Aqp10.2b paralogs. Oocytes that express the European eel Aqp10.2bs were incubated in a solution containing 5 mM of ^15^N_2_-urea for 3 or 6 min. The amount of ^15^N_2_-urea absorbed by the oocytes was then quantified using LC–MS. Values for the ^15^N-labeled urea content are presented as mean ± SD and also compared to oocytes expressing Aqp10.2b paralogs and control oocytes. Statistical significances (*P* values) were calculated by one-way analysis of variance (ANOVA) followed by Dunnett's test (****P < 0.0001, **P < 0.01, and *P < 0.05). All samples are listed in Table S7.

In a previous study by MacIver et al. (2009), the European eel Aqpe (Aqp10.2b1) expressed in *Xenopus* oocytes exhibited permeability to water, glycerol, and urea. However, our swelling assay showed that the European eel Aqp10.2b1, expressed in *Xenopus* oocytes, has low permeability to urea. MacIver et al. performed uptake experiments using [^14^C]urea in solution containing 5 mM urea (2009), while a solution containing 180 mM urea was used in the swelling assay. Therefore, to analyze urea permeability at low concentrations of urea, we examined the uptake of ^15^N_2_-urea by oocytes expressing Aqp10.2 paralog. Using mass spectrometry, we detected the uptake of ^15^N_2_-urea by oocytes at the pmol/oocyte level. ^15^N_2_-urea was nearly undetectable in control oocytes, but Aqp10.2b1 oocytes took up urea, although the difference was not statistically significant (Fig. 3). The oocytes expressing Aqp10.2b2 and Aqp10.2b3 absorbed more urea (Fig. 3). These results suggested that Aqp10.2b2 and Aqp10.2b3 were more permeable to urea than Aqp10.2b1 at both low and high urea concentrations.

### Solute and Water Permeabilities of Aqp10.2b in Anguilliformes Species, other than *Anguilla* Species

Anguilliformes species, other than *Anguilla* species, have a single Aqp10.2b. Here, we analyzed the activity of Aqp10.2b in Kaup's arrowtooth eel and European conger using a swelling assay. For comparison, we analyzed Aqp10.2b in the Indo-Pacific tarpon. Oocytes expressing Aqp10.2b of these three species showed significant volume gain and increased *P_water_* in a hypoosmotic solution (Fig. 2), confirming that these Aqp10s act as water channels in the plasma membrane of oocytes.

In an iso-osmotic solution containing glycerol, all oocytes expressing Aqp10.2b of these three species showed significant volume gain and increased *P_glycerol_* (Fig. 2), indicating that these Aqp10s are permeable to glycerol. In an iso-osmotic solution containing urea and boric acid, all oocytes expressing Aqp10.2b of these three species showed weak or no increase in *P_urea_* and *P_boric acid_* (Fig. 2).

### Effects of *Y205G* Mutation on the Urea and Boric Acid Permeabilities of European Eel Aqp10.2b1

**Fig. 4.**
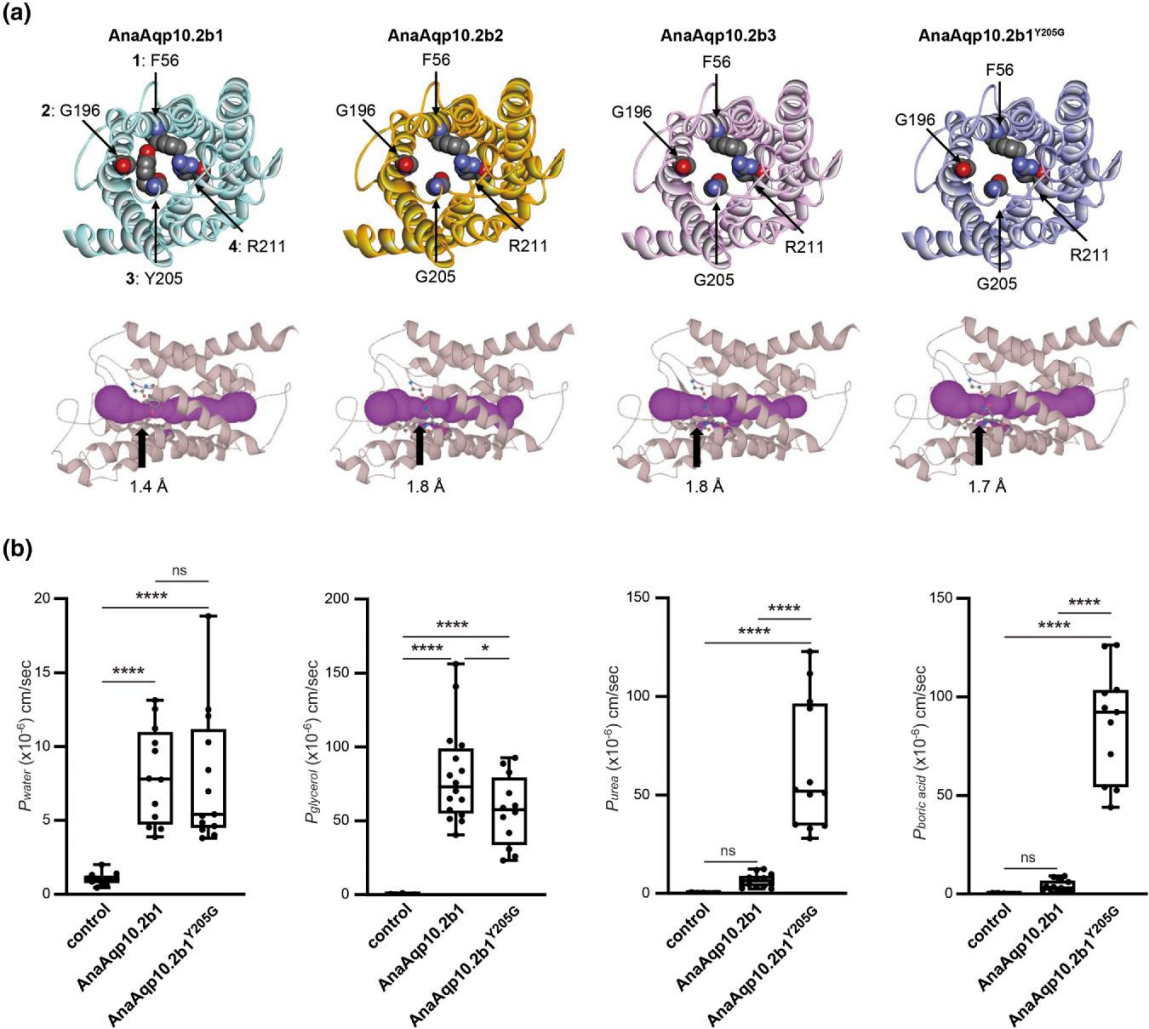
Structural and functional characterization of European eel Aqp10.2bs. a) Model subunit structures of AnaAqp10.2b1, 10.2b2, 10.2b3, and 10.2b1^Y205^ and their MOLE (pore) predictions. The ar/R region residues are shown as sphere at the top (positions 1 to 4 are indicated in AanAqp10.2b1). Each ar/R selectivity region is indicated by an arrow and its pore radius is shown below it at the bottom. b) Activities of European eel Aqp10.2b1 and its mutant. Water and solute permeabilities of AanAqp10.2b1 and AanAqp10.2b1^Y205G^. Water (*P*_water_), glycerol (*P*_glycerol_), urea (*P*_urea_), and boric acid (*P*_boric acid_) permeabilities of oocytes expressing AanAqp10.2b1 and its mutant compared to those of control oocytes. Data were plotted using box-and-whisker plots (median: 25% to 75%ile; 1.5× interquartile). Statistical significance was assessed via one-way ANOVA, followed by Tukey's post-hoc test (****P < 0.0001 and *P < 0.05). All samples are listed in Tables S5 and S6. Aan, *Anguilla anguilla*.

Activity analyses of European eel Aqp10 paralogs revealed that European eel Aqp10.2b2 and b3 underwent a Y-to-G substitution at position 3 of the ar/R selectivity filter and acquired solute selectivity with high *P_urea_* and *P_boric acid_*. Previously, we demonstrated that the sum of the molecular weights of the amino acid residues in the ar/R selectivity filter and pore size influence urea and boric acid permeabilities (Nagashima et al. 2025b). Our prior work also demonstrated that mutant of Aqp10.2bs with a Y-to-G change at position 3 had a larger pore and enabled transport of urea and boric acid. In accordance with this observation, the estimated pore radius of AanAqp10.2b2 and AanAqp10.2b3 was 1.8 Å, which is larger than the 1.4 Å estimated pore radius of AanAqp10.2b1 (Fig. 4a). Similarly, the AanAqp10.2b1 Y to G mutant at position 3 exhibited a comparatively substantial estimated pore radius of 1.7 Å (Fig. 4a). Therefore, we confirmed whether this Y-to-G substitution directly modified the solute selectivity by expressing European eel Aqp10.2b1^Y205G^ in *Xenopus* oocytes and analyzing its activity.

Oocytes expressing Aqp10.2b1^Y205G^ showed significant volume gain and increased *P_water_* in the hypoosmotic solution (Fig. 4b), confirming that this mutant acted as a water channel in the plasma membrane of oocytes. In an iso-osmotic solution containing glycerol, urea, and boric acid, oocytes expressing Aqp10.2b1^Y205G^ showed significant volume gain and increased *P_glycerol_*, *P_urea_*, and *P_boric acid_* (Fig. 4b), suggesting that the *Y205G* mutation increased *P_urea_* and *P_boric acid_* of European eel Aqp10.2b1.

### Tissue Distribution of Aqp10.2b1 and b2 in Japanese Eel

**Fig. 5.**
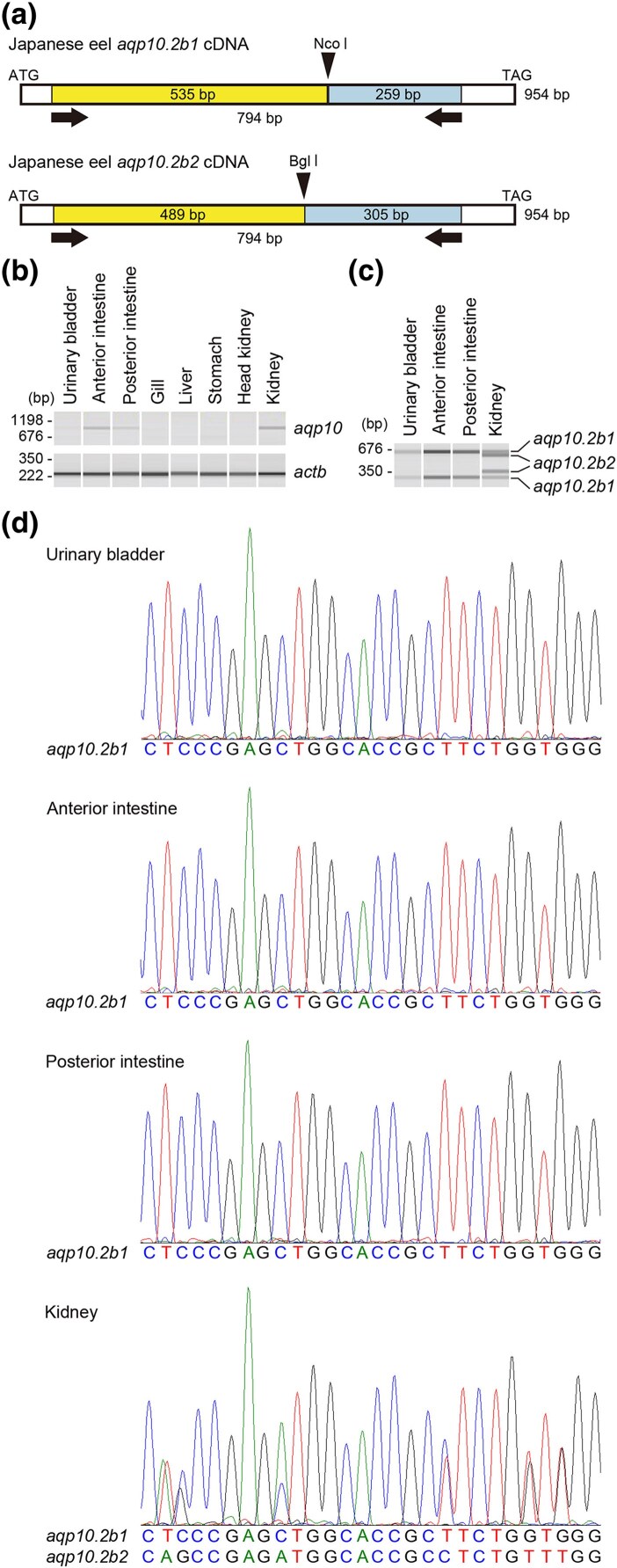
Tissue distribution of *aqp10.2b1* and *aqp10.2b2* in Japanese eel. a) Primer design for Japanese eel *aqp10*. Schematic representation of the positions of the two *aqp10* cDNA paralogs in Japanese eel are shown with the primers used for the RT-PCR. The primers (arrows) were designed within the conserved region between the two *aqp10* cDNA paralogs to amplify both. The PCR products include paralog-specific restriction enzyme sequences, NcoI and BglI (arrowheads). Numbers indicate the size in base pairs (bp) of the PCR products with or without restriction enzyme digestion. b) Tissue distribution of *apq10* paralogs in Japanese eel reared in fresh water. PCR products were analyzed by a microchip electrophoresis without restriction enzyme digestion. The generation of pseudo-gel images of the PCR products was facilitated by the microchip electrophoresis system, and the β-actin gene (*actb*) was utilized as an internal control. The whole images of the gels are shown in Fig. S6. c) Expression of *apq10* paralogs in freshwater-reared Japanese eels. PCR products were digested with NcoI and BglI and then similarly analyzed by microchip electrophoresis. d) Direct sequencing of the PCR products. The PCR products derived from the urinary bladder, anterior intestine, posterior intestine, and kidney of Japanese eel shown in (b) were analyzed by direct sequencing.

Martinez et al. (2005a, 2005b) analyzed the expression of European eel Aqpe (Aqp10.2b1) in the intestine and kidney using northern blotting, and Kim et al. (2010) analyzed the expression of Japanese eel Aqp10 (Aqp10.2b1) in several tissues using RT-PCR. The *aqp10.2b* paralogs in European eels have a high nucleotide sequence identity (96% to 99%) in the coding region, suggesting that northern analysis may not be able to distinguish between them. The RT-PCR analysis performed by Kim et al. (2010) used a set of primers that matched 100% with Japanese eel *aqp10.2b1* and *aqp10.2b2*. Therefore, the results of Martinez et al. (2005a, 2005b) and Kim et al. (2010) are likely to detect the total expression levels of all *aqp10.2b* paralogs in European and Japanese eels, respectively. To analyze the tissue distribution of *aqp10.2b* paralogs, we performed semiquantitative RT-PCR on various tissues of the Japanese eel that are readily available in Japan. The nucleotide sequences of the coding regions of *aqp10.2b1* and *aqp10.2b2* in the Japanese eel were 97% identical, and primers could not be designed in order to distinguish between them. Therefore, a set of primers that amplify both genes was designed (Fig. 5a). Semiquantitative RT-PCR revealed the expression of *aqp10.2b* in the urinary bladder, posterior intestine, anterior intestine, and kidney of the Japanese eel (Fig. 5b). The PCR products derived from *aqp10.2b1* contained NcoI sites, whereas those derived from *aqp10.2b2* contained BglI sites (Fig. 5a). Therefore, the PCR products were digested with NcoI and BglI and analyzed by electrophoresis. Digestion products derived solely from *aqp10.2b1* were detected in the urinary bladder, posterior intestine, and anterior intestine, whereas digestion products derived from both *aqp10.2b1* and *aqp10.2b2* were detected in the kidneys (Fig. 5c). Direct sequencing of the PCR products revealed that the sequence derived from *aqp10.2b1* was present in the PCR products derived from the urinary bladder, posterior intestine, and anterior intestine, whereas both *aqp10.2b1* and *aqp10.2b2* sequences were detected in the PCR products derived from the kidney (Fig. 5d). These results indicate that the urinary bladder, posterior intestine, and anterior intestine of the Japanese eel express only *aqp10.2b1*, whereas the kidney expresses both *aqp10.2b1* and *aqp10.2b2*.

### Relatively Rapid Nonsynonymous Substitution (dN) Rates in *Anguilla aqp10.2b2* and *aqp10.2b3*

**Fig. 6.**
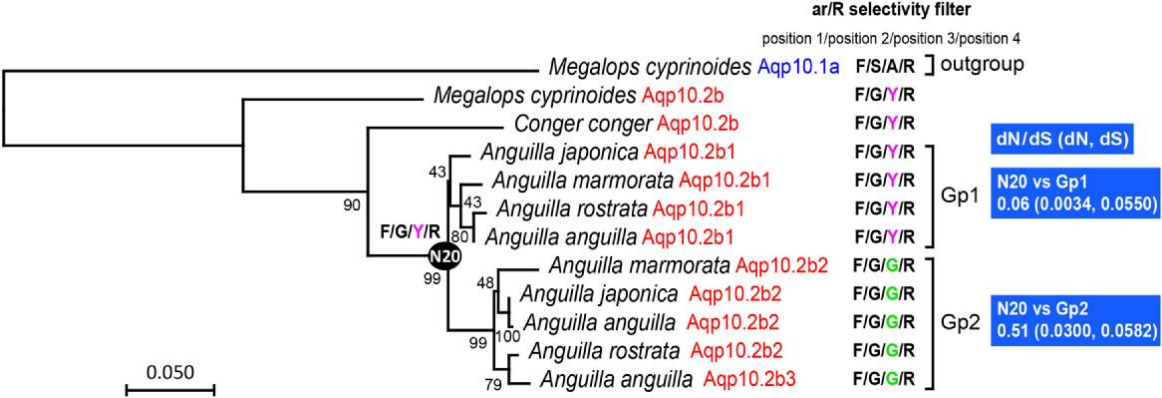
Rapid evolution of Aqp10.2b2 and Aqp10.2b3 in eels. Phylogenetic analyses of the nucleotide sequences of Aqp10s in Elopomorpha species. Numbers indicate the bootstrap values, and scale bar represents the genetic distance of nucleotide substitutions per site. Average Jukes–Cantor (JC) distances of Aqp10-coding regions within the same or among different groups are shown in the phylogenetic tree. Variants were estimated using the bootstrap method with 500 replicates. Nucleotide sequences used for analysis are shown in Fig. S5. dN, nonsynonymous substitutions per site; dS, synonymous substitutions per site.

Activity analyses results of European eel Aqp10 paralogs revealed that the function of European eel Aqp10.2b1 is similar to that of Aqp10.2b in other Anguilliformes species, and is similar to the typical activity of Aqp10.2s in ray-finned fishes (Imaizumi et al. 2024), whereas Aqp10.2b2 and b3 acquired new functions after tandem duplication of the genes encoding these proteins. We hypothesized that the dN rates in *aqp10.2b2* and *aqp10.2b3* may be altered compared to that in *aqp10.2b1*. To calculate the dN and synonymous substitution (dS) rates, we analyzed the nucleotide sequences of *aqp10.2b* paralogs in four *Anguilla* species: giant mottled, Japanese, American, and European eels. A phylogenetic tree was constructed based on the alignment of the nucleotide sequences, as shown in Fig. 6. The *Anguilla* Aqp10.2b paralogs with Y at position 3 in the ar/R selectivity filter and those with G at the same position formed two different clades, which are indicated as groups 1 (Gp1) and 2 (Gp2), respectively, as shown in Fig. 6. Using this alignment, we constructed the most probable sequences for the root of *Anguilla* Aqp10 s as ancestral sequences, N20 in Fig. 6. The amino acid residue at position 3 in the ar/R selectivity filter of the estimated ancestral sequence, N20, was Y. These results suggest that in the ancestral *Anguilla* species, one of the tandem duplicates of *aqp10.2b* underwent a Y-to-G substitution, which is conserved in *aqp10.2b* paralogs in Gp2 (Fig. 6).

dN and dS were calculated for N20, Gp1, and Gp2 based on the Nei–Gojobori (NG) method (Nei and Gojobori 1986), and the results are shown in Fig. 6. The dN between N20 and Gp2 was 0.030, which was approximately nine times higher than that between N20 and Gp1 (0.0034). dS between N20 and Gp2 was 0.055, which was not significantly different from that between N20 and Gp1 (0.058). The dN/dS ratio between N20 and Gp2 was 0.51, whereas that between N20 and Gp1 was 0.06. These results indicated high dN values in Aqp10.2b2 and Aqp10.2b3 of *Anguilla* species.

## Discussion

**Fig. 7.**
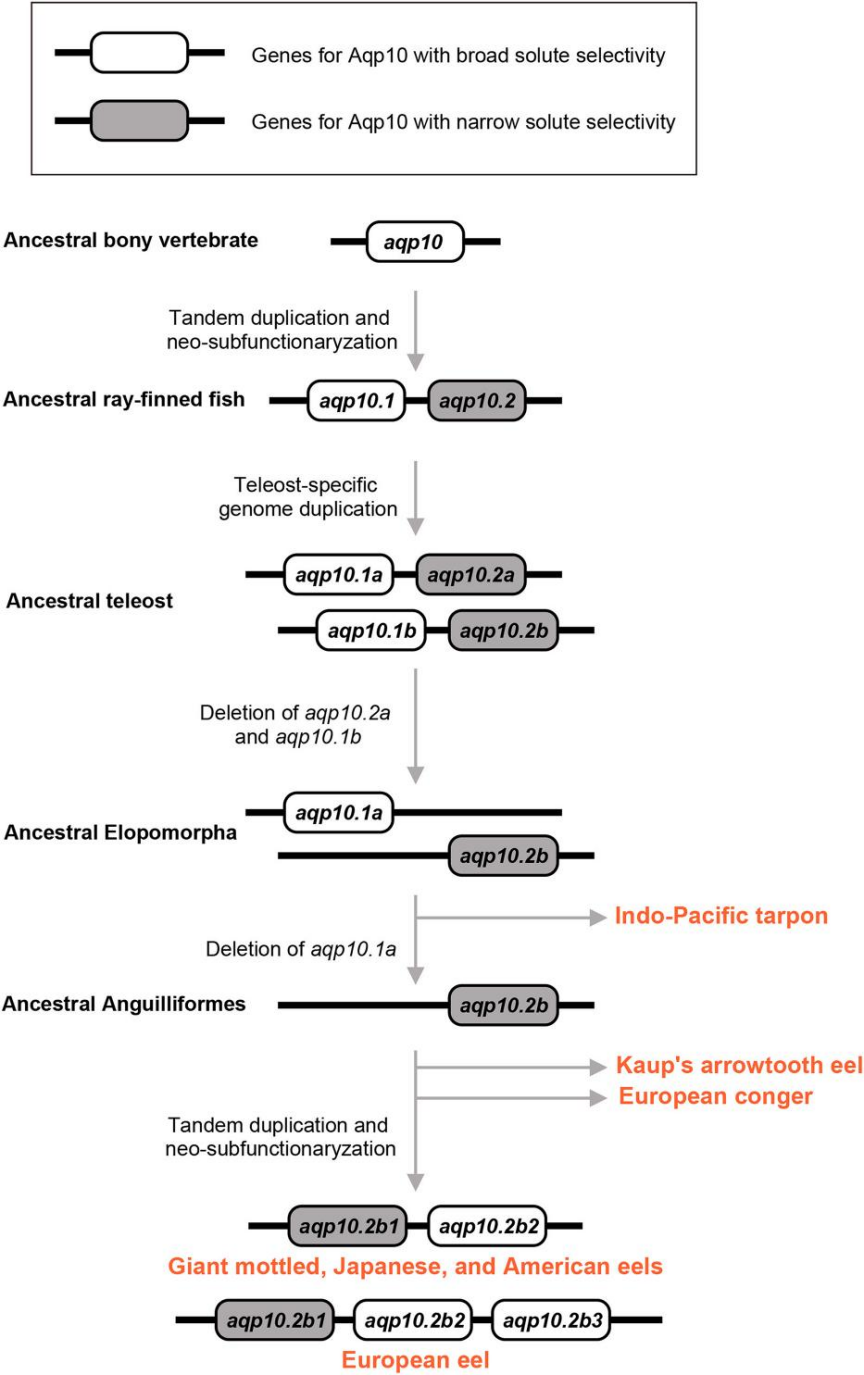
Proposed timeline of the loss and gain of Aqp10 paralogs with broad solute selectivity in *Anguilla* species. A schematic representation of the evolutionary history leading to the identification of *aqp10* paralogs in European eel compared with that in other *Anguilla* species, European conger, Kaup's arrowtooth eel, and Indo-Pacific tarpon, is shown. The names of extant species are indicated. The analyzed and estimated solute selectivities of the protein product of each gene are shown on white (broad solute selectivity) or gray (narrow solute selectivity) backgrounds.

The common ancestor of ray-finned fish acquired an additional *aqp10* paralog through tandem duplication (Yilmaz et al. 2020). Owing to amino acid substitutions, these paralogs have diversified in solute permeability, resulting in two main types: one with broad solute permeability and the other with glycerol-specific permeability (Imaizumi et al. 2024). As most teleosts retain at least one ortholog from each of these ancient paralogs, the functional diversity in *aqp10* appears to be crucial for their physiology. These genes make excellent models for studying gene duplication and neo-subfunctionalization. Notably, *Anguilla* species have a unique evolutionary history for these genes. Their common ancestor lost all *aqp10.1*-derived paralogs; however, new paralogs have been reacquired within the *Anguilla* species through recent tandem duplications of *aqp10.2b*. In the present study, we investigated these newly duplicated genes. By comparing the amino acid sequences and solute permeabilities of the three Aqp10.2b paralogs in European eels, we found that the eels have reacquired an *aqp10* paralog with broad solute selectivity (*aqp10.2b2* and *aqp10.2b3*). This compensated for the ancestral loss of the Aqp10.1-derived broad permeability through recent gene duplications (Fig. 7).

Analysis of the amino acid sequences of eel Aqp10.2b paralogs revealed that one of the four amino acid residues forming the ar/R selectivity filter was substituted from Y to G in two of three European eel paralogs. Previous studies have reported that the two aromatic amino acid residues at positions 1 and 3 of the four sites forming the ar/R selectivity filter are important for reducing urea and boric acid permeability, but not for glycerol permeability, in Aqp10 (Nagashima et al. 2025b). Aromatic amino acid residues have relatively large molecular weights, and the two aromatic amino acid residues in the ar/R selectivity filter reduce the pore size, limiting the transport of solutes. The results of the activity analysis and structural modeling showed that the European eel Aqp10.2b paralog with Y at position 3 in the ar/R selectivity filter, Aqp10.2b1, exhibited narrow transport solute selectivity and possessed an relatively small estimated pore radius similar to those of other Aqp10.2s, whereas the Y-to G-substituted paralogs, Aqp10.2b2 and b3, exhibited high urea and boric acid transport activity and possessed a relatively large estimated pore radius similar to that of Aqp10.1s. These findings imply that recent gene duplication in ancestral *Anguilla* species resulted in the acquisition of an Aqp10 paralog with broad solute selectivity, similar to the previously lost Aqp10.1.

Analysis of the substitution rates of *aqp10* in Anguilliformes showed a relatively low dN/dS ratio of ∼0.06 between the estimated ancestral *Anguilla aqp10.2b* sequence N20 and *Anguilla aqp10.2b1*. In contrast, the dN/dS ratio between N20 and *Anguilla aqp10.2b* paralogs with high urea and boric acid permeability (eg European eel *aqp10.2b2* and *aqp10.2b3*) was ∼0.5. In general, adaptive evolution is indicated by a dN/dS ratio greater than 1. In the present study, the dN/dS ratio of ∼0.5, in the *Anguilla aqp10.2b2* and *aqp10.2b3*, was less than 1, but this can be considered as a result of simultaneously observing the purifying selection of amino acid residues that contribute to the structure essential for channel formation and adaptive selection that altered transport solute selectivity.

In this study, we clarified that *Anguilla* species reacquired a type of Aqp10 with broad transport substrate selectivity, which they had previously lost. However, this result raises a new question. Given that other Anguilliformes lineages, such as Kaup's arrowtooth eel and the European conger, appear to survive well without these *aqp10* paralogs, what selective advantage do these duplications confer on *Anguilla* species? Most ray-finned fish species typically possess at least one gene derived from both *aqp10.1* and *aqp10.2* (Yilmaz et al. 2020), but the order Anguilliformes is a rare exceptions to this rule. Although it is plausible that other aquaglyceroporins compensate for the transport functions of lost *aqp10.1* paralogs in other anguilliform species, the specific benefits of these duplications in *Anguilla* remain unclear. Nevertheless, it is conceivable that possessing extra Aqp10 paralogs offers an evolutionary advantage by enabling the precise regulation of transport pathways through altered expression or functional differentiation. Further research is needed to determine these advantages and their physiological implications precisely.

Mammalian Aqp10 is thought to contribute to glycerol transport in intestinal epithelial cells and adipocytes (Mobasheri et al. 2004; Gotfryd et al. 2018). In teleost fish, the primary expression site of *aqp10* is the intestine, as demonstrated in both European and Japanese eels (Martinez et al. 2005a; Kim et al. 2010), zebrafish (Tingaud-Sequeira et al. 2010), Atlantic salmon (Tipsmark et al. 2010), and Japanese pufferfish (Kumagai et al. 2023), suggesting that one of the primary roles of fish Aqp10 is the transport of water and solutes in the intestine (Takei 2021). The intestines of zebrafish and Japanese pufferfish express both *aqp10.1a* and *aqp10.2b* (Tingaud-Sequeira et al. 2010; Kumagai et al. 2023). In the Japanese eel, this study demonstrated that the intestine expresses only one *aqp10* paralog, *aqp10.2b1*, which may be considered a characteristic of the Anguilliformes. The role of Aqp10 in freshwater and seawater acclimation remains unclear (Madsen et al. 2015; Takei 2021). Aqp10 expression in the kidney has been observed in zebrafish (Tingaud-Sequeira et al. 2010) and European eels (Martinez et al. 2005a), and was also observed in Japanese eels in this study. Unlike other organs, the kidneys of the Japanese eel express both *aqp10.2b1* and *aqp10.2b2*. Currently, the kidney of the Japanese eel is the only organ in which *aqp10.2b2* expression has been confirmed in *Anguilla* species. Thus, Aqp10.2b1 and Aqp10.2b2 are important targets for the physiological analysis of functional differentiation.

Yilmaz et al. (2020) revealed that tandem duplication of *aqp* genes occurred many times at various ages during vertebrate evolution and diversification. The major ones include the tandem duplication of *aqp9/13-like* in Hyperoartia, *aqp3* and *aqp10* in cartilaginous fishes, *aqp10* in ray-finned fishes, *aqp3b* in Gadidae teleosts, *aqp3a* in Cichlidae teleosts, *aqp10.2b* in Anguillidae teleosts, *aqp10.1a* in Cyprinodontiformes teleosts, *aqp7* in Homininae, *aqp10* in select amphibian, reptile, and avian lineages. However, physiological significance of these tandem duplications remains unclear. Based on the findings of our previous report, we analyzed *aqp10* paralogs in ray-finned fish and showed that their biochemical activity can be estimated using the four amino acid residues forming the ar/R selectivity filter (Nagashima et al. 2025b). Our approach can help in predicting the activity and solute selectivity of aquaglyceroporins of other species using the four amino acid residues forming the ar/R selectivity filter estimated from the genomic data of various species.

## Materials and Methods

### Synteny and Phylogenetic Analyses

The presence and number of *aqp10.1* and *aqp10.2* orthologs were determined via synteny analysis. Genome databases of Elopomorpha species, Indo-pacific tarpon (*Megalops cyprinoides*; GCA_013368585.1), roundjaw bonefish (*Albula glossodonta*; GCA_019788955.1) (Pickett et al. 2022), Kaup's arrowtooth eel (*Synaphobranchus kaupii*; GCA_029718625.1) (Parey et al. 2023), giant moray (*Gymnothorax javanicus*; GCA_029692085.1) (Parey et al. 2023), European conger (*Conger conger*; GCA_963514075.1) (Parey et al. 2023), Japanese eel (*A. japonica*; GCA_025169545.1) (Wang et al. 2022), American eel (*A. rostrata*; GCA_018555375.3) (Pavey et al. 2017), and European eel (*A. Anguilla*; GCA_013347855.1) (Henkel et al. 2012), were browsed using the Ensembl genome browser (https://www.ensembl.org) (Martin et al. 2023) and National Center for Biotechnology Information genome data viewer (https://www.ncbi.nlm.nih.gov/genome/gdv/) (Rangwala et al. 2021). The *aqp10.1* and *aqp10.2* loci were manually examined. Accession numbers of all genes are listed in Table S1. The *aqp10.2* orthologs in giant mottled eel (*A. marmorata*; GCA_901111315.1) (Barth et al. 2020) were identified via tBLASTn analysis using the genome database.

The amino acid sequences and accession numbers of the Aqp10s of Elopomorpha species were collected from the GenBank/EMBL/DDBJ or Ensembl genome browsers (Table 1). Giant mottled eels Aqp10.2b1 and b2 and Japanese eel Aqp10.2b2 were not annotated; thus, they were manually annotated and registered as third-party annotations (TPA) in the DDBJ (accession numbers: BR002453, BR002454, and BR002452, respectively). We also manually annotated the roundjaws Aqp10.1a and Aqp10.2b and registered them as TPA to the DDBJ (BR002456 and BR002457, respectively). The amino acid sequences were aligned with ClustalW software (Chenna et al. 2003) and ESPript (Robert and Gouet 2014) was used to produce graphical display of the results. The evolutionary history was inferred using the Maximum Likelihood method and the JTT matrix-based model (Jones et al. 1992). The tree with the highest log likelihood (−2,830.67) is shown. The percentages of trees in which the associated taxa were clustered together are shown below the branches. The initial trees for the heuristic search were obtained automatically by applying the Neighbor-Join and BioNJ algorithms to a matrix of pairwise distances estimated using the JTT model and selecting the topology with a superior log likelihood value. The tree was drawn to scale with branch lengths measured as the number of substitutions per site. This analysis involved 10 amino acid sequences. The final dataset contained 332 positions. Evolutionary analyses were conducted using MEGA11 software (Tamura et al. 2021).

### Dot Plot Analysis

*aqp10* loci were compared among European, Japanese, and American eels via dot plot analysis using the EMBOSS dotmatcher program (https://www.ebi.ac.uk/jdispatcher/emboss) (Madeira et al. 2024) with a window size of 20 and a threshold score of 70. All analyzed genomic regions are presented in Table S2.

### Molecular Clock Analysis

All nucleotide sequences encoding Aqp10s listed in Table 1 and Table S3 were aligned with those of using ClustalW software (Chenna et al. 2003), and sites containing gaps were deleted manually without shifting the reading frame (Fig. S4). A time tree was generated using multiple alignment data and the RelTime method (Tamura et al. 2012). Divergence time points for all branching points in the topology were calculated using the Maximum Likelihood method and Tamura–Nei model (Tamura and Nei 1993). The estimated log likelihood value of the topology shown was –3,644.82. The tree was drawn to scale with branch lengths measured as the relative number of substitutions per site. Six nucleotide sequences were analyzed. The final dataset contained 976 positions. Evolutionary analyses were conducted using MEGA11 software.

### Expression Vector Construction and Site-Directed Mutagenesis

Full-length cDNAs with 15-bp sequences complementary to the ends of the linearized pGEMHE of Aqp10s from the European eel, Kaup's arrowtooth eel, European conger, and Indo-Pacific tarpon and a site-directed mutagenesis sequence (AanAqp10.2b1Y205G) were chemically synthesized (Eurofins Genomics, Tokyo, Japan), as previously described (Imaizumi et al. 2024; Nagashima et al. 2025b). The cDNAs were subcloned into pGEMHE (Liman et al. 1992) using the NEBuilder HiFi DNA Assembly Master Mix (New England Biolabs, Ipswich, MA, USA) and sequenced.

### Expression of Aqp10 in *Xenopus* Oocytes

Plasmids were linearized with NotI (Takara Bio, Shiga, Japan), and capped RNAs (cRNAs) were transcribed in vitro using the T7 Message Machine kit (Thermo Fisher Scientific, Waltham, MA, USA). *Xenopus laevis* oocytes were dissociated with collagenase, as previously described (Romero et al. 1998), and injected with 50 nL of water or a solution containing 0.5 ng/nL cRNA (25 ng/oocyte) using the Nanoject-II injector (Drummond Scientific, Broomall, PA, USA). Oocytes were incubated in the OR3 medium at 16 °C and observed for 4 to 5 d after injection. The OR3 medium (1 L) consisted of 0.7% w/v powdered Leibovitz L-15 medium with L-glutamine (Thermo Fisher Scientific), 50 mL of 10,000 U penicillin, 10,000 U streptomycin solution in 0.9% NaCl (Nacalai Tesque Inc., Kyoto, Japan), and 5 mM HEPES (pH 7.50) (Romero et al. 1998). Osmolality was adjusted to 200 mOsmol/kg using NaCl powder. All animal protocols and procedures were conducted in accordance with the National Institutes of Health Guide for the Care and Use of Laboratory Animals and approved by the Institutional Animal Experiment Committee of the Tokyo Institute of Technology.

### *Xenopus* Oocyte Swelling Assay

Oocyte swelling was monitored using a stereomicroscope (SZX9; Olympus, Tokyo, Japan) equipped with a charge-coupled device camera (DS-Fi2; Nikon, Tokyo, Japan) as previously described (Ushio et al. 2022; Kumagai et al. 2023; Imaizumi et al. 2024). The oocyte volume was calculated assuming a spherical geometry. Oocytes incubated with ND96 (approximately 200 mOsmol/kg) were transferred to 2-fold diluted ND96 (approximately 100 mOsmol/kg) for water transport assays. For glycerol, urea, and boric acid transport assays, oocytes were transferred to an isotonic solution containing ND96 supplemented with 180 mM glycerol, urea, or boric acid instead of NaCl and adjusted to an osmolality of approximately 200 mOsmol/kg. Water permeability (*P*_water_) was calculated from the osmotic swelling data and the molar volume of water (*V_w_* = 18 cm^3^/mol) as follows (Preston et al. 1992): *P*_water_ = [*V_o_* × *d*(*V*/*V_o_*)/*dt*]/[*S* × *V_w_* × (osm_in_ − osm_out_)], where *S* is the initial oocyte surface area. Solute permeability (*P*_solute_) was calculated from the swelling data, total osmolality of the system (osm_total_ = 200 mOsmol/kg), and solute gradient (sol_out_ − sol_in_) as follows (Carbrey et al. 2003): *P*_solute_ = osm_total_ × [*V_o_* × *d*(*V*/*V_o_*)/*dt*]/[*S* × (sol_out_ − sol_in_)]. The water, glycerol, urea, and boric acid transport activities of each Aqp10 strain were evaluated using oocytes from the same animal, and the experiment was repeated using at least three frogs. The list of plot data is shown in Tables S4 and S5. Quantitative data are represented as the mean ± standard deviation in Table S6. *P_water_* and *P_solute_* values were compared between the Aqp10-expressing and control oocytes, and statistical significance was assessed using one-way analysis of variance, followed by the Holm–Sidak test or Tukey's test using GraphPad Prism software (version 8; GraphPad, San Diego, CA, USA) to visualize the results in box plots.

### Uptake Assays for ^15^N-labeled Urea by Oocyte Expressing aqp10

Oocytes expressing European eel Aqp10.2b paralogues or control oocytes injected with water were placed in ND96 medium containing 5 mM ^15^N-labeled urea (^15^N_2_-urea; Sigma–Aldrich, St. Louis, MO, USA). Each oocyte was washed twice with ND96 and the wash solution was removed after 10 oocytes were collected in a tube. To frozen *Xenopus* oocytes 500 μL methanol containing 50 μM morpholinoethanesulfonic acid (MES) and 100 μM methionine sulfone were added as internal standards, then this was homogenized with BioMasher II and 250 μL ultrapure water was added. To 600 μL of the suspension, 400 μL of chloroform was added, mixed and centrifuged at 12,000 rpm for 5 min at 4 °C. The 450 μL supernatant was collected and concentrated by centrifugation for 30 min. To this sample, 300 μL of ultrapure water was added and ultrafiltrated with Amicon Ultra 3k (Merk Millipore, Darmstadt, Germany) at 13,000 rpm for 30 min at 4 °C, and all the filtrate was collected. The samples were frozen at −80 °C for approximately 15 min, lyophilized for 6 h, and dissolved in 66 μL of ultrapure water for liquid chromatography (LC)–Mass Spectrometry (MS) analysis.

The list of plot data is shown in Table S7. Quantitative data for oocytes from at least three frogs are presented as mean ± SD. Values for ^15^N-labeled urea content were compared between oocytes expressing European eel Aqp10.2bs and control oocytes. Statistical significance (*P* values) was calculated by one-way analysis of variance (ANOVA) followed by Dunnett's test using GraphPad Prism software (version 8; GraphPad, San Diego, CA, USA) to visualize the results in bar plots.

### Chromatographic Conditions for Liquid Chromatography Triple Quadrupole Mass Spectrometry System

An LC-triple quadrupole MS system (LCMS-8030; Shimadzu, Kyoto, Japan) was used for 15N-urea quantification after extraction. 15N-urea was separated on a PFPP column (Discovery HS F5, 2.1 mm I.D. × 150 mm L, 3 μm; Supelco, Bellefonte, PA, USA). Water (solvent A) and acetonitrile (solvent B) containing 0.1% (v/v) formic acid were used as mobile phases. The gradient was programmed to hold at 0% B for the first 2 min, rise from 0% to 25% B for 3 min, from 25% to 35% B for 6 min, from 35% to 95% B for 4 min, and finally held at 95% B for 5 min at a flow rate of 250 μL/min. Fragments were detected by electrospray ionization in the positive ion mode. Mass spectra (63.00 > 19.05 *m*/*z*) were obtained in MRM scan modes. Data were acquired and analyzed using the LabSolutions software (Shimadzu).

### Structural Modeling of AanAqp10s

For the pore analysis, structural models of AnaAqp10.2b1, 10.2b2, 10.2b3 and 10.2b1^Y205G^ were first generated using the AlphaFold 3 server (Abramson et al. 2024). Then, according to previous studies (Ushio et al. 2022; Nagashima et al. 2025b), pore analysis was performed on the TMD core part (residues 14 to 261) of each model structure using MOLE online (Pravda et al. 2018).

### Semiquantitative RT-PCR and Sequence Analysis

Total RNA from freshwater-acclimated Japanese eels (Nakada et al. 2005) was used in this study. First-strand complementary DNA was synthesized from 5 μg of total RNA using the SuperScript IV First-Strand Synthesis System (Thermo Fisher Scientific) with oligo(dT) primers. The samples were then analyzed by RT-PCR, as previously described (Tran et al. 2006; Motoshima et al. 2023) using GoTaq Green Master Mix (2×; Promega, Madison, WI, USA) and a set of primers: 5′-ccgagtgtttgggagtctacgtcttg-3′ and 5′-tagagccagtttgttgtccacggtt-3′. These primers were designed to amplify Japanese eel *aqp10.2b1* and *aqp10.2b2*. β-Actin (*actb*) was similarly analyzed as an internal control using a set of primers: 5′-ccttcctgggtatggagtcctg-3′ and 5′-cggagtatttgcgctcaggtg-3′ (Wong et al. 2016).

The PCR conditions were as follows: initial denaturation at 94 °C for 2 min; 33 cycles of 94 °C for 15 s (denaturation); 52 °C for 30 s (annealing); 72 °C for 1 min (extension); and a final extension at 72 °C for 7 min. Five microliter of the PCR mixture was two times diluted and loaded onto a microchip electrophoresis system for DNA/RNA analysis (MCE-202 MultiNA, Shimadzu) using a DNA-2500 reagent kit (Shimadzu) in accordance with the manufacturer's instructions. Electrophoresis results were analyzed using MultiNA Viewer software (Shimadzu).

The PCR products of Japanese eel *aqp10* cDNA were purified using the Fast Gene Gel/PCR extraction kit (Nippon Genetics, Tokyo, Japan) and sequenced directly with one of the primers used for *aqp10* cDNA amplification using a Big Dye Terminator ver3.1 (Applied Biosystems, Waltham, MA, USA) and a 3730xl DNA Analyzer (Applied Biosystems). Sequence data were visualized using the MEGA11 software.

### Restriction Enzyme Digestion of PCR Products Derived From Japanese Eel *aqp10.2b1* and *aqp10.2b2* cDNAs

Under the same aforementioned conditions, 25 μL of the 38-cycle PCR products of Japanese eel *aqp10* cDNA were purified using the Fast Gene Gel/PCR extraction kit (Nippon Genetics). The eluates were digested with 15 U NcoI (Nippon Gene, Tokyo, Japan) and 12 U BglI (Nippon Gene) in the H buffer at 37 °C for 3 h, followed by re-purification using the Fast Gene Gel/PCR extraction kit. Aliquots (5 μL) of the eluates were diluted 2-fold and analyzed using the microchip electrophoresis system and DNA-2500 reagent kit as described above.

### Calculation of the Nucleotide Substitution Rate

Protein coding sequences of Aqp10s listed in Table 1 were aligned with those of using ClustalW software (Chenna et al. 2003), and the sites containing gaps were deleted manually without shifting the reading frame as described above.

Ancestral states were inferred using the Maximum Likelihood method (Nei and Kumar 2000) and Tamura–Nei model (Tamura and Nei 1993). The tree shows a set of possible nucleotides (states) at each ancestral node based on their inferred likelihood at Site 1. The rates among the sites were treated as uniform (uniform rate option). The analysis included 12 nucleotide sequences. The final dataset contained 603 positions. Evolutionary analyses were conducted using MEGA11 software.

Protein-coding sequences of Aqp10s listed in Table 1 were aligned with those of predicted ancestral sequences using ClustalW software (Fig. S5), sites containing gaps were manually deleted without shifting the reading frame, and the alignment was used to calculate the nucleotide substitution rates.

The mean distance values between groups for dN and dS were calculated based on the NG method (Nei and Gojobori 1986) using an alignment composed of 13 sequences and 522 positions with the MEGA11 software. Standard errors were computed using the bootstrap method with 500 replicates.

## Supplementary Material

evaf169_Supplementary_Data

## Data Availability

Data generated/analyzed in this study are available in this article and Supplementary Materials. All identified sequences have been deposited into GenBank under the accession numbers BR002452 to BR002454 and BR002456 to BR002457 as indicated in Materials and Methods section.
