# Supplementary material for: Loss and Gain of Aqp10 Paralogs With Broad Solute Selectivity in Anguillid Eels
Source: Genome Biol Evol. 2025 Sep 12;17(10):evaf169. doi: 10.1093/gbe/evaf169 (PMC12492003; doi:10.1093/gbe/evaf169)
Supplement: evaf169_Supplementary_Data [file evaf169_supplementary_data.pdf]

Supplementary Table S1. Synteny analyses of *aqp10* in Indo-pacific tarpon, roundjaw bonefish, and Anguilliformes

Species	Chromosome, scaffold, etc.	Direction	Annotated genes in each region						
Indo-pacific tarpon (*Megalops cyprinoides*)	Chromosome 10	Plus	c1orf43<<br>XP_036395072.1	ubap2l><br>XP_036394589.1	si:dkey-92i15.4><br>XP_036393795.1	hax1><br>XP_036394568.1	**aqp10.1a><br>XP_036394569.1**	atp8b2><br>XP_036394812.1	il6r><br>XP_036395749.1
Roundjaw bonefish (*Albula glossodonta*)	JAFBMS010000018.1	Minus	c1orf43<<br>KAG9345380.1	ubap2l><br>KAG9345379.1	si:dkey-92i15.4><br>KAG9345378.1		**aqp10.1a><br>KAG9345377.1<br>BR002456**	atp8b2><br>KAG9345376.1	il6r><br>KAG9345375.1
Kaup's arrowtooth eel (*Synaphobranchus kaupii*)	Chromosome 7	Minus	c1orf43<<br>KAJ8354720.1	ubap2l><br>KAJ8354719.1		hax1><br>KAJ8354718.1		atp8b2><br>KAJ8354717.1	il6r><br>KAJ8354716.1
Giant moray (*Gymnothorax javanicus*)	Chromosome 1	Plus	c1orf43<<br>KAJ8290021.1	ubap2l><br>KAJ8290022.1	si:dkey-92i15.4><br>KAJ8290023.1			atp8b2><br>KAJ8290024.1	il6r><br>KAJ8290025.1
European conger (*Conger conger*)	Chromosome 1	Minus	c1orf43<<br>XP_061102897.1	ubap2l><br>XP_061089925.1	si:dkey-92i15.4><br>XP_061118192.1			atp8b2><br>XP_061089909.1	il6r><br>XP_061104589.1
Giant mottled eel (*Anguilla marmorata*)	no data								
Japanese eel (*Anguilla japonica*)	Chromosome 1	Minus	c1orf43<<br>JAZDXO010000001.1<br>REGION:<br>70098759..70104182	ubap2l><br>JAZDXO010000001.1<br>REGION:<br>70065317..70097368	si:dkey-92i15.4><br>JAZDXO010000001.1<br>REGION:<br>70052913..70059624			atp8b2><br>JAZDXO010000001.1<br>REGION:<br>69994684..70038342	il6r><br>JAZDXO010000001.1<br>REGION:<br>69982669..69993693
American eel (*Anguilla rostrata*)	Chromosome 1	Minus	c1orf43<<br>XP_064209827.1	ubap2l><br>XP_064209813.1	si:dkey-92i15.4><br>XP_064209780.1			atp8b2><br>XP_064209746.1	il6r><br>XP_064209768.1
European eel (*Anguilla anguilla*)	Chromosome 1	Minus	c1orf43<<br>XP_035265727.1	ubap2l><br>XP_035260557.1	si:dkey-92i15.4><br>XP_035260525.1			atp8b2><br>XP_035288547.1	il6r><br>XP_035261967.1
Indo-pacific tarpon (*Megalops cyprinoides*)	Chromosome 21	Minus		ubap2l><br>XM_036555172.1		hax1><br>XM_036555570.1	**aqp10.2b><br>XP_036372080.1**		
Roundjaw bonefish (*Albula glossodonta*)	JAFBMS010000001.1	Plus		ubap2l><br>KAG9355291.1			**aqp10.2b><br>KAG9355292.1<br>BR002457**		
Kaup's arrowtooth eel (*Synaphobranchus kaupii*)	Chromosome 21	Minus		ubap2l><br>KAJ8335136.1		hax1><br>KAJ8335135.1 (N)	**aqp10.2b><br>KAJ8335135.1 (C)**		
Giant moray (*Gymnothorax javanicus*)	Chromosome 5	Plus		ubap2l><br>KAJ8273542.1 (N)		hax1><br>KAJ8273542.1 (C)	**aqp10.2b><br>KAJ8273543.1**		
European conger (*Conger conger*)	Chromosome 9	Plus		ubap2l><br>XP_061110189.1		hax1><br>XP_061111355.1	**aqp10.2b><br>XP_061110608.1**		
Giant mottled eel (*Anguilla marmorata*)	CAAKNA010030492.1,<br>CAAKNA010024719.1	Plus					**aqp10.2b1><br>BR002453**	**aqp10.2b2><br>BR002454**	
Japanese eel (*Anguilla japonica*)	Chromosome 8	Minus		ubap2l><br>JANEZO010000008.1<br>REGION:<br>48291650..48327284		hax1><br>JANEZO010000008.1<br>REGION:<br>48280939..48287699	**aqp10.2b1><br>JANEZO010000008.1<br>REGION:<br>48265945..48278577<br>(BAH89255.1)**	**aqp10.2b2><br>JANEZO010000008.1<br>REGION:<br>48249330..48258254<br>BR002452**	
American eel (*Anguilla rostrata*)	Chromosome 8	Minus		ubap2l><br>XP_064201517.1		hax1><br>XP_064201558.1	**aqp10.2b1><br>XP_064204597.1**	**aqp10.2b2><br>XP_064204595.1**	
European eel (*Anguilla anguilla*)	Chromosome 8	Minus		ubap2l><br>XP_035285411.1		hax1><br>XP_035283697.1	**aqp10.2b1><br>XP_035284278.1**	**aqp10.2b2><br>XP_035286386.1**	**aqp10.2b3><br>XP_035286385.1**

Supplementary Table S2. Genomic regions analyzed by dot plot analysis.

Species and gene	Region	Accession no.
Japanese eel *aqp10.2b1* , *aqp10.2b2*	47400000..47560000	JANEZO010000008.1
European eel *aqp10.2b1* , *aqp10.2b2* , *aqp10.2b3*	48090000..48290000	fAngAng1.pri (GCF_013347855.1)
American eel (EN2019) *aqp10.2b1* , *aqp10.2b2*	49130000..49280000	ASM1855537v3 (GCF_018555375.3)
European eel *aqp10.2b2*	47527000..47536000	fAngAng1.pri (GCF_013347855.1)
European eel *aqp10.2b3*	47454000..47463000	fAngAng1.pri (GCF_013347855.1)
American eel (EN2019) *aqp10.2b2*	49240500..49249500	ASM1855537v3 (GCF_018555375.3)
American eel (LakeOntario-01-2011) *aqp10.2b2*	13000..22000	LTYT01013103.1

Supplementary Table S3. Coding and amino acid sequences of aquaglyceroporins (Aqp10s) analyzed/identified in this study

Species	Protein name in this study	Accession no.	Coding sequence	Amino acid sequence
Indo-pacific tarpon (*Megalops cyprinoides*)	Aqp10.1a	XM_036538676.1	atggacaaactgaaacagaagctgagtgtgaggaataatctgaccagggagtgtatgggggagtttctgg<br>gcacatttgttctgctgttgtttggttgtgcagcaggggcccagataaagaccagtcaagagacaaagggc<br>cagttcctctccggcaacatggccttctctgtgggcgtcatgtctgccatgtacctctgtgggggtgtctctg<br>gggctcatctgaaccctgctgtgtctctcagcttctgcgtcttaggacggttgccctggagcaagctgctgc<br>catactccctctcccagattctgggagcctacatggcctctggagttgtcttcatgacatactatgatgccatc<br>atgcattacagtgggggaaacttgacagtatttggacccagggaaactgcctccatttttgccacgtacccat<br>ctgactatctgtctcttgggagcagcttccttgaccaggttgtcggcacggctatgctgttactctgtatactgc<br>ccctggatgacaagctaaacaaccctgccccctctgccttgatcccccccattgtggcagtggtggtcttgg<br>gaatatctatgtccatgtccgctaactgtggggcggccatcaacccggccagggacctggggcccagact<br>gttcacacttactgcaggctgggggactgaggttttcacgtgctataactactggttctgggttcctttggtag<br>cgcctatgctgggggctgtagttgggaccggattctacctggtgttcatccgatggcacctgcctgacccg<br>aatgctgccaggagcagaactgccaccatggagtctcagcctggggaacagaaactgccacctgtccgt<br>gaaaccagaacaaccagctttaaaatagagacaggtaaacagttctga	mdklkqklsvrnnltrecmgeflgtf<br>vlllfgcaagaqiktsqetkgqflsgn<br>mafsvgvmsamylcggvsgahln<br>pavslsfcvlgrlpwskllpyslsqil<br>gaymasgvvfmtyydaimhysgg<br>nltvfgpretasifatypsdylslgssfl<br>dqvvgtamlllcilplddklnnpaps<br>alippivavvvlgismsmsancgaa<br>inpardlgprlftltagwgtevftcyn<br>ywfwvplvapmlgavvgtgfylvf<br>irwhlpdpnaarsrtatmesqpgeq<br>klppvretrttsfkietgkqf
	Aqp10.2b	XM_036516187.1	atggagtcattgctgaggagatgccgcatcaggagcagtttggtgagagagtgtttagctgagtgcctggg<br>tgtctatgtcctgattctgtttggctgtggggccgttgcccaggtgacaacgtctgagaacagcaagggaca<br>ctacatatccatcaacctgggctttgcgttgggcactacctttggtatctatgtctccagaggcgtgtcaggg<br>gcccatctgaacccggcagtgaccctcagcctttgtgtcctcggcagacacccctggagaaacctgccctt<br>ctacgtcttcttccaggtgctgggggccttcctcgctgcggcaaccattgccctgcagtactacgacgccat<br>aatgcactatagcaacgggcagttgaccgtgactggactgaccgccaccgctgggatctttgctacttacc<br>ctgcagattacctgagcctgtggagtggctttgtggaccaggtgattggcacaggagcattgctggtgtgta<br>tcttggccctgggtgaccacagaaacacccctgtcccacctggattggcaccggttctagtaggcttggtg<br>gtcttggtgatcgggatctccatgggctccaactgtggatacgccctgaacccagcccgagacctgggac<br>cacggatctacacctacatctcaggctggggtgacgaggtcttcaggtgggctgggggaggttggtggtg<br>ggtgccgctggttgctccctgtgtgggggcccttgtagggacgatggtctatgagcttctcattgaggtaca<br>ccacccagagacagagcttcacccggaagatgaccctgagcgtcaaaccatcgacaacaagcaggccc<br>tggagctggagggggtggaactggacctgaatggcctcaagagctgccccaaagaggggcaagaaga<br>caggaagaatgtgagggggactatggaggaggagggctgtccagacaagttttga	mesllrrcrirsslvreclaeclgvyvl<br>ilfgcgavaqvttsenskghyisinlg<br>falgttfgiyvsrgvsgahlnpavtlsl<br>cvlgrhpwrnlpfyvffqvlgaflaa<br>atialqyydaimhysngqltvtgltat<br>agifatypadylslwsgfvdqvigtg<br>allvcilalgdhrntpvppglapvlvg<br>lvvlvigismgsncgyalnpardlg<br>priytyisgwgdevfrwagggww<br>wvplvapcvgalvgtmvyelliev<br>hhpetelhpeddperqtidnkqalel<br>egveldlnglkscpkegqedrknvr<br>gtmeeegcpdkf
Roundjaw bonefish (*Albula glossodonta*)	Aqp10.1a	BR002456	atggacaaagttaggaggactctcagagtgacagactccctgacaagggagtgcatgggggagtttttgg<br>gcacctttgttctcctgtgttttggctgttcagctgcagcacaggtaaagactagtcaggagacaaagggcc<br>agttcctctccgtcaacttggccttctctgtgggtgtgatgtctgccatgtacctctgtggaggagtctcgggg<br>gctcacctgaacccggctgtgtctctgagtttctgtgctctgggacggctggcctggggcaagctgctgcc<br>gtattgcctggcccagctgctgggggcctacacagcttctgccctcgtcttctttgtgtaccacgatgctataa<br>tgcaattcagtgggggaaacctgactgtgtttggacccactgaaacggcctctatctttgcaacttacccctc<br>tgagtatctgtctctagggtccagcttcctggaccaggtggttggcacagcaatgttattgctgtgtatactgc<br>ccctggatgaggagagaaacaaccctgccccctctgccctgatcccacccattgtggctggggtggtcct<br>ggggatctccatgtccatgtctgctaactgtggggcagctatcaatcctgcccgggacctggggccccgtc<br>tcttcacactaactgcaggctgggggaccgaagtgtttacgtgctataactactggttttgggtacccttgttg<br>gcaccaatgctagggagtgttgtgggaactggattttacctgatcttcataaagtggcaccttcctgacccag<br>accctgccaggagcagaactggaactctggaaaatgagtctgggggacagaaatgggaatcaaccgttg<br>aaagaggaacaacaagttttaaaatagagaccagcaaacacttttga	mdkvrrtlrvtdsltrecmgeflgtfv<br>llcfgcsaaaqvktsqetkgqflsvnl<br>afsvgvmsamylcggvsgahlnpa<br>vslsfcalgrlawgkllpyclaqllga<br>ytasalvffvyhdaimqfsggnltvf<br>gptetasifatypseylslgssfldqvv<br>gtamlllcilpldeernnpapsalipp<br>ivagvvlgismsmsancgaainpar<br>dlgprlftltagwgtevftcynywfw<br>vpllapmlgsvvgtgfylifikwhlp<br>dpdparsrtgtlenesggqkwestv<br>ergttsfkietskhf
	Aqp10.2b	BR002457	atggactcagtatttaggagggtccgtgtcaggagtagtctggtcagagaatgcctggctgagtgtcttgga<br>gtctatgtcatgattgtgtttggttgtgggtccgttgcccaggtgacaacctctgagaacagcaaagggcact<br>acatatccatcaacctgggctttgcgcttggcaccacctttggcatctatgtctcccgaggcgtatcagggg<br>cccacctgaaccccgctgtgaccctcagcctgtgtgtcctgggcaggcacccctggaggaagctgccctt<br>ctacatcttcttccaggtgctgggggccttcctcgccgctgccaccattgccctgcagtactacgatgctatt<br>atgcacttcagcagcggccagctgatggtcacagggccgactgcaaccgccgggatctttgctacttacc<br>ctgcagagtacctcagcttgtggggcggcttcatggaccaggtgattgggacgggcatgcttctggtgtgc<br>gtcctggctgtgggggaccacaggaacaccccgtcccgccgggactgtcgccagtcctggtgggcctg<br>gtggtcctagtgattgggctgtccatgggctccaactgcggctatgccctgaacccagccagagacctgg<br>ggcccagactgtacagctacatcgcaggctggggagacgaggtcttcagggctggaggagggtggtgg<br>tgggtccctctggttgccccctgtgtgggggcgctggtggggacaatgacatacgagctcctaatcgagg<br>cacaccatccagaggtgggcctcgacccagatctgtgccaaaccacggtcaacaagatgaccgtggagc<br>tggagggggtgcagctgcaggagaattcgtccaagagctcccccagcaaaaggctggaggacaagaaa<br>cccacacaagggacgatggtcgaacaggaggggtag	mdsvfrrvrvrsslvreclaeclgvy<br>vmivfgcgsvaqvttsenskghyis<br>inlgfalgttfgiyvsrgvsgahlnpa<br>vtlslcvlgrhpwrklpfyiffqvlga<br>flaaatialqyydaimhfssgqlmvt<br>gptatagifatypaeylslwggfmd<br>qvigtgmllvcvlavgdhrntpvpp<br>glspvlvglvvlviglsmgsncgyal<br>npardlgprlysyiagwgdevfrag<br>ggwwwvplvapcvgalvgtmtye<br>llieahhpevgldpdlcqttvnkmtv<br>elegvqlqenssksspskrledkkpt<br>qgtmveqeg
Kaup's arrowtooth eel (*Synaphobranchus kaupii*)	Aqp10.2b	KAJ8335135.1 (amino-acid residues 281–599)	atggactctgtatttcggagggtgcgactccggagccgtctgctcagagagtgcctggcggagtgtctgg<br>gggtctacgtcataatcgtgtttggctgcgggaccgtcgcccaggtgacgacctctgagaacagcaaggg<br>gcattacctgtccatcaacctgggctttgccctcggcaccaccttcggggtctacgtctctcgaggcgtgtc<br>aggagctcacctgaaccccgctgtcacgctcagcctgtgcatgctgggaaggcacccttggaggaagct<br>gcccttctacgtcttctgccaggtgtttgggggcttcctcggtgccgctactgtcgccttgcagtactacgatg<br>ccattatgcacttcagcaacggccagctgactgtgacaggaccgactgccaccgctggaattttcgccacc<br>taccctgcagattacctcagtctgtggggcggctttgtggaccaggtaatcgggactgcgatgctactggtg<br>tgcatcctgtgcgtgggagactgcaggaacaccgccgtctcaccagagctggcaccggttctagtgggcc<br>tggtcgtcctggcgatcgggatgtccatgggcgtcaactgcgggtacgccctgaacccggctcgagactt<br>cgggccccggctctacagctacctcgccggctggggagaccaagtgttctgggcgggggggagggtggt<br>ggtgggtccctctcgttgcaccctgtgtcggggcgattgtggggtccgtggcgtacgagcttctaatcgag<br>gcgcaccatccagatctgggcctcgacccggaaaaggcagcgcagtgccaaaccgtggagaacaagct<br>ggctctggagctagagggggtacaattggacctgaatgcccccaagggttgccccaacgaagggcagg<br>aaggcgggaaggctgtgagagggacgtcgggcggggaggaggggtag	mdsvfrrvrlrsrllreclaeclgvyvi<br>ivfgcgtvaqvttsenskghylsinlg<br>falgttfgvyvsrgvsgahlnpavtls<br>lcmlgrhpwrklpfyvfcqvfggfl<br>gaatvalqyydaimhfsngqltvtg<br>ptatagifatypadylslwggfvdqv<br>igtamllvcilcvgdcrntavspelap<br>vlvglvvlaigmsmgvncgyalnp<br>ardfgprlysylagwgdqvfwagg<br>gwwwvplvapcvgaivgsvayel<br>lieahhpdlgldpekaaqcqtvenkl<br>alelegvqldlnapkgcpnegqegg<br>kavrgtsggeeg
Giant moray (*Gymnothorax javanicus*)	Aqp10.2b	KAJ8273543.1	atggagtccgtgttccgaagagtgcgaatcaggagccttctcatcagggagtgcctggcggagtgtttggg<br>cgtctacgtcctaatagtgtttggatgtggggctgtagcccaggtgaccacctctgaaaacaccaagggac<br>attacctgtcaatcaacctgggattcgctctgggcaccaccttcggggtttacgtctctcgcggcgtgtcagg<br>ggctcacctgaaccccgcagtcacactcagcctgtgcattctgggaaggcacccctggaagtctctgccat<br>tctacgtcttcttccagatctttggggccttccttgcggccgccactgtggctttgcagtactacgatgccatta<br>tgcacttcacaaacggggagctgactgtgaaagggcccacagccaccgctgggatttttgcaacatacccct<br>gcagattacctccatctatggagcggctttgtggaccaggtgattgggaccggcatgctgctactgtgcatc<br>ctggctataggggaccgcagaaacacctcagtacccccgaaatggcggcagtccttgttggtctggccg<br>tcttggcaatcggggtgtccatgggggttaactgcggctacgcgctaaaccccgcccgtgacctgggacc<br>caggctgtacacctacatcgcaggctgggggggaccaggtattccgggctggagaaggctggtggtgggt<br>gcccctggtcgccccctgtgttggggcagttgtgggatccctggtatatgtgctcctaatagaagctcatca<br>cccagaactggaccttgaccaggaaaaggctgaccaatgtcaaaccatggacaacaagctggccctgga<br>gctggagggggtacaactggacctaaatgccccccagggctgtaccaacggaggacaggaaggcaag<br>aaggtgatgagggggactaggggcgaggaggggtag	mesvfrrvrirsllireclaeclgvyvl<br>ivfgcgavaqvttsentkghylsinlg<br>falgttfgvyvsrgvsgahlnpavtls<br>lcilgrhpwkslpfyvffqifgaflaa<br>atvalqyydaimhftngeltvkgpta<br>tagifatypadylhlwsgfvdqvigt<br>gmlllcilaigdrrntsvppemaavl<br>vglavlaigvsmgvncgyalnpard<br>lgprlytyiagwgdqvfragegww<br>wvplvapcvgavvgslvyvllieah<br>hpeldldqekadqcqtmdnklalel<br>egvqldlnapqgctnggqegkkv<br>mrgtrgeeg
European conger (*Conger conger*)	Aqp10.2b	XM_061254624.1	atggattccgtgttcaggagagttcggatcaggaacgttctgctccggcagtgcctggcggagtgtttggg<br>agtctacatcctgatcgtgtttggctgtggggccgttgcccaggtgaccacctctgagaacagcaagggcc<br>agtacctgtccatcaacctgggcttcgccctgggggccaccttcggggtctacgtctcgcagggcgtgtca<br>ggagctcatctgaaccccgctgtcaccctcagcctctgcatcctgggaaggcacccctggaggagcctgc	mdsvfrrvrirnvllrqclaeclgvyi<br>livfgcgavaqvttsenskgqylsinl<br>gfalgatfgvyvsqgvsgahlnpavt<br>lslcilgrhpwrslpfyvffqvfgafla

			ccttctacgtcttcttccaggtgtttggggccttcctcgcggccgccaccgtcgccttgcagtactacgacgc<br>catcatgcacttcagcagtgggcagctcactgtgacaggaccaacagcgaccgctggaattttcgccactt<br>accctgcagattacctgaacctgtggagcggcttcgcagaccaggtcatcggcacggcgatgctgctggt<br>gtgcgtgctggctgtgggggaccgcaggaacacccccgtcccgccagagctggggccgctcctcgtgg<br>gcctgatcgtcctggtgatcggcatttccatgggcgccaactgcggctacgccctcaacccggcccggga<br>cctgggcccccgcctgtacagctacgtcgcgggctggggagaccaggtgttctgggcgggcggaggct<br>ggtggtgggtccctctggttgccccctgtgtaggggcgctggtggggtctgcggtgtacgtgttcctaatcg<br>aggcgcaccacccggatctgtccctcgacgcggagcgggctgatcagtgccacaccgtggacaacaag<br>ctggccctggagctggagggggtagaactggacccaaatacccccaagggctgccccaccgaggggc<br>aggaagccaagaagggggagcaggggtag	aatvalqyydaimhfssgqltvtgpt<br>atagifatypadylnlwsgfadqvig<br>tamllvcvlavgdrrntpvppelgpl<br>lvglivlvigismgancgyalnpard<br>lgprlysyvagwgdqvfwagggw<br>wwvplvapcvgalvgsavyvflie<br>ahhpdlsldaeradqchtvdnklale<br>legveldpntpkgcptegqeakkge<br>qg
Giant mottled eel (*Anguilla marmorata*)	Aqp10.2b1	BR002453	atggactccgtgttcaggagagtccgaatccagagccgtctgctcaggcagtgcctggccgagtgtttgg<br>gagtctacgtcttgattgtgtttggctgtggggccgtcgcccaggtgacgacctctgagaacaccaagggg<br>cagtacctgtccatcaacctagcctttgccctaggcaccacctttggagtctacgtctctcgaggcgtgtcag<br>gagctcacctgaaccctgcagtcaccctcagcctgtgcatgctgggaaggcacccttggaggactctgcc<br>cttctatgtcttcttccaggtgtttggggccttccttgccgctgccaccgtcgccttgcagtactacgatgccat<br>tatgcacttcagcaatgggcagctgacagtaacaggaccgactgccaccgctggaattttcgccacttacc<br>ctgcagattacctcagtctgtggagtggctttgttgaccaggtgattgggactggcatgctgctggtgtgcat<br>cctggctgtgggggaccgcagaaacacccgcatccctcccgagctggcaccgcttctggtgggcctggtt<br>gtcctggtgatcggggtgtccatgggcgtcaactgtggctacgccctcaacccagcccgagacctgggg<br>ccccggctgtacagctatatcgcaggctggggagagcaggtgttctgg	mdsvfrrvriqsrllrqclaeclgvyv<br>livfgcgavaqvttsentkgqylsinl<br>afalgttfgvyvsrgvsgahlnpavtl<br>slcmlgrhpwrtlpfyvffqvfgafl<br>aaatvalqyydaimhfsngqltvtg<br>ptatagifatypadylslwsgfvdqv<br>igtgmllvcilavgdrrntrippelapl<br>lvglvvlvigvsmgvncgyalnpar<br>dlgprlysyiagwgeqvfw
	Aqp10.2b2	BR002454 (5' end incomplete)	gtgtttggctgtgcggccgccgcccaggtgacgacctctgagaacaccaagggggcagtacctgtccatca<br>acctagcctttgccctaggcaccaccttcggagtctacgtctctcgaggcgtgtcaggagctcacctgaac<br>cctgcagtcaccctcagcctgtgcatgctgggaaggcacccttggaggactctgcccttctatgtcttcttcc<br>aggtgtttggggcctttctcgctgctgccactgtcgccttgcagtactatgatgccattatgcacttcagcaat<br>gggcaactgactgtaacaggaccgactgccaccgctgcaattttcgccacttaccctgcagattacctcagt<br>ctgtggagtggctttgttgaccaggtgattgggactggcatgctgctggtgtgcatcctggctctggaagac<br>cgcaggaacacctccatcccagccgagatggcaccgcctctgtttggcctggttgtcctggtgatcgggat<br>gtccatgagcgtcaactgtggcggcgccctcaacccggcccgagacctggggccccggctgtacagcta<br>tatcgcaggctggggagagcaagtgttctgggcgggggggaggatggtggtgggtccctctggttgctccc<br>tgtgtcggagcgctggtggggtccgtagtgtatgtgctcctgattgaggcacatcacccagagctggacct<br>ccacctggaaaaggccgaccagtgccaaaccgtggacaacaaactggctctagagctggagggggtcg<br>agctggacctgaattcccccaagggctgccccaacgaagggcaggagggcaagaaggctgcaagtgg<br>gaagggtgaggaggggtag	vfgcaaaaqvttsentkgqylsinlaf<br>algttfgvyvsrgvsgahlnpavtlsl<br>cmlgrhpwrtlpfyvffqvfgaflaa<br>atvalqyydaimhfsngqltvtgpta<br>taaifatypadylslwsgfvdqvigt<br>gmllvcilaledrrntsipaemapplf<br>glvvlvigmsmsvncggalnpard<br>lgprlysyiagwgeqvfwagggw<br>wwvplvapcvgalvgsvvyvllie<br>ahhpeldlhlekadqcqtvdnklale<br>legveldlnspkgcpnegqegkkaa<br>sgkgeeg
Japanese eel (*Anguilla japonica*)	Aqp10.2b1	AB378503.1	atggactccgtgttcaggagagtccgaatccagagccgtctgctcagacagtgcctggccgagtgtttggg<br>agtctacgtcttgatcgtgtttggctgtggggccgttgcccaggtgacgacgtctgagaacaccaaggggc<br>agtacctgtccatcaacctaggctttgccctaggcaccaccttcggagtctacgtctctcgaggcgtgtcag<br>gagctcacctgaaccccgcagtcaccctcagcctgtgcatgctgggaaggcacccttggaggactctgcc<br>cttctatgtcttcttccaggtgtttggggccttccttgccgctgccaccgtagccttgcagtactacgatgccat<br>tatgcacttcagcaatgggcagctgacagtaacaggaccgactgccaccgctggaattttcgccacatacc<br>ctgcagattacctcagtctgtggagtggctttgtggaccaggtgattgggactggtatgctgctggtgtgcat<br>cctggctgtgggggaccgcaggaacacccgcatccctcccgagctggcaccgcttctggtgggcctggt<br>tgtcctggtgatcggggtgtccatgggcgtcaactgcggctacgccctcaacccggcccgagacctggg<br>gccccggctgtacagctatatcgcgggctggggagagcaggtgttctgggcgggggggaggatggtggt<br>gggtccctctggttgctccctgtgtcggagcgctggtggggtccgtagtgtatgtgctcctgattgaggcac<br>atcacccagagctggacctccacctggaaaaggccgaccagtgccaaaccgtggacaacaaactggctc<br>tagagctggagggggtcgagctggacctgaattcccccaagggctgccccaacgaagggcaggaggg<br>caagaaggctgcaagtgggaagggtgaagaggggtag	mdsvfrrvriqsrllrqclaeclgvyv<br>livfgcgavaqvttsentkgqylsinl<br>gfalgttfgvyvsrgvsgahlnpavtl<br>slcmlgrhpwrtlpfyvffqvfgafl<br>aaatvalqyydaimhfsngqltvtg<br>ptatagifatypadylslwsgfvdqv<br>igtgmllvcilavgdrrntrippelapl<br>lvglvvlvigvsmgvncgyalnpar<br>dlgprlysyiagwgeqvfwaggg<br>wwwvplvapcvgalvgsvvyvlli<br>eahhpeldlhlekadqcqtvdnklal<br>elegveldlnspkgcpnegqegkka<br>asgkgeeg
	Aqp10.2b2	BR002452	atggactccgtgttcaggagagtccgaatccagagccgtctgctcagacagtgcctggccgagtgtttggg<br>agtctacgtcttgatcgtgtttggctgtgcggccactgcccaggtgacgacctctgagaacaccaaggggc<br>agtacctgtccatcaacctaagctttgccctaggcaccaccttcggagtctacgtctctcgaggcgtgtcag<br>gagctcacctgaaccccgcagtcaccctcagcctgtgcatgctgggaaggcacccttggaggactctgcc<br>cttctatgtcttcttccaggtgtttggggccttccttgccgctgccaccgtcgccttgcagtactacgatgccat<br>tatgcacttcagcaatgggcaactgactgtaacaggaccgactgccaccgctgcaattttcgccacttaccc<br>tgcagattacctcagtctgtggagtggctttgttgaccaggtgattgggactggcatgctgctggtgtgcatc<br>ctggctgtggaagaccgcaggaacacccgcatcccagccgagatggcaccgcctctgtttggcctggttg<br>tcctggtgatcggaatgtccatgagcgtcaactgcggcggcgccctcaacccggcccgagacctggggc<br>cccggctgtacagctatatcgcgggctggggagagcaggtgttctgggcgggggggaggatggtggtgg<br>gtccctctggttgctccctgtgtcggagcgctggtggggtccggagtgtatgtgctcctgattgaggcacat<br>cacccagagctggacctccacctggaaaaggccgaccagtgccaaaccgtggacaacaaactggctcta<br>gagctggagggggtcgagctggacctgaatttccccaagggctgccccaacgaagggcaggagggca<br>agaaggctgcaagtgggaagggtgaagaggggtag	mdsvfrrvriqsrllrqclaeclgvyv<br>livfgcaataqvttsentkgqylsinls<br>falgttfgvyvsrgvsgahlnpavtls<br>lcmlgrhpwrtlpfyvffqvfgafla<br>aatvalqyydaimhfsngqltvtgpt<br>ataaifatypadylslwsgfvdqvigt<br>gmllvcilavedrrntripaemappl<br>fglvvlvigmsmsvncggalnpar<br>dlgprlysyiagwgeqvfwaggg<br>wwwvplvapcvgalvgsgvyvlli<br>eahhpeldlhlekadqcqtvdnklal<br>elegveldlnfpkgcpnegqegkka<br>asgkgeeg
American eel (*Anguilla rostrata*)	Aqp10.2b1	XM_064348527.1	atggactccgtgttcaggagagtccggatccagagccgtctgctcagacagtgcctggccgagtgtttgg<br>gagtctacgtcttgatcgtgtttggctgtggggccgtcgcccaggtgacgacctctgagaacaccaaggg<br>gcagtacctgtccatcaacctagcctttgccctaggcaccacctttggagtctatgtctctcgaggcgtgtca<br>ggagctcacctgaaccccgcagtcaccctcagcctgtgcatgctgggaaggcacccttggaggactctgc<br>ccttctatgtcttcttccaggtgtttggggccttccttgccgctgccaccgtcgccttgcagtactacgatgcc<br>attatgcacttcagcaatgggcaactgactgtaacaggaccgactgccaccgctggaattttcgccacttac<br>cctgcagattacctcagtctgtggagtggctttgtggaccaggtgattgggactggcatgctgctggtgtgc<br>atcctggctgtgggggaccgcaggaacacccgcatccctcctgagctggcaccgcttctggtgggcctg<br>gttgtcctggtgatcgggatatccatgggcgtcaactgtggctacgccctcaacccggccagagacctgg<br>ggccccggctgtacagctatatcgcgggctggggagagcaagtgttctgggcgggggggaggatggtgg<br>tgggtccctctggttgctccctgtgtcggagcgctggtggggtctgtagtgtatgtgctcctgattgaggcac<br>atcacccagagctggaccttcacctggaaaaggctgaccagtgccaaaccgtggacaacaaactggctct<br>agagctggagggggtcgagctggacctgaattcccccaagggctgccccaacgaagggcaggagggc<br>aagaaggctgcaagtgggaagggtgaggaggggtag	mdsvfrrvriqsrllrqclaeclgvyv<br>livfgcgavaqvttsentkgqylsinl<br>afalgttfgvyvsrgvsgahlnpavtl<br>slcmlgrhpwrtlpfyvffqvfgafl<br>aaatvalqyydaimhfsngqltvtg<br>ptatagifatypadylslwsgfvdqv<br>igtgmllvcilavgdrrntrippelapl<br>lvglvvlvigismgvncgyalnpar<br>dlgprlysyiagwgeqvfwaggg<br>wwwvplvapcvgalvgsvvyvlli<br>eahhpeldlhlekadqcqtvdnklal<br>elegveldlnspkgcpnegqegkka<br>asgkgeeg
	Aqp10.2b2	XM_064348525.1	atggactccgtgttcaggagagtccggatccagagccgtctgctcagacagtgcctggccgagtgtttgg<br>gagtctacgtcttgatcgtgtttggctgtggggccgctgcccaggtgacaacctctgagaacaccaagggg<br>cagtacctgtccatcaacctaggctttgccctaggcaccacctttggagtctacgtctctcgaggcgtgtcag<br>gagctcacctgaatcctgcagtcaccctcagcctgtgcatgctgggaaggcacccttggaggactctgcc<br>cttctatgtcttcttccaggtgtttggggccttccttgccgctgccactgtcgccttgcagtactatgatgccatt<br>atgcacttcagcaatgggcaactgactgtaacaggaccgactgccaccgctgcaattttcgccacttaccct<br>gcagattacctcagtctgtggagtggcttcgttgaccaggtgattgggactggcatgctgctggtgtgcatc<br>ctggccgtggaagaccgcaggaacacccgcatcccagccgagatggcaccgcctctgtttggcctggtt<br>gtcctggtgatcgggatgtccatgagcgtaaactgcggcggcgccctcaacccggcccgagacctgggg<br>ccccggctgtacagctatatcgcgggctggggagagcaggtgttctgggcagggggaggatggtggtg<br>ggtccctctggttgctccctgtgttggagcgctggtggggtccgtagtgtatgtgctcctgattgaggcacat<br>cacccagagctggacctccacctggaaaaggccgaccagtgccaaaccgtggacaacaaactggctcta<br>gagctggagggggtcgagctggacctgaattcccccaagggctgccccaacgaagggcaggagggca<br>agaaggctgcaagtgggaagggtgaggaggggtag	mdsvfrrvriqsrllrqclaeclgvyv<br>livfgcgaaaqvttsentkgqylsinl<br>gfalgttfgvyvsrgvsgahlnpavtl<br>slcmlgrhpwrtlpfyvffqvfgafl<br>aaatvalqyydaimhfsngqltvtg<br>ptataaifatypadylslwsgfvdqvi<br>gtgmllvcilavedrrntripaemap<br>plfglvvlvigmsmsvncggalnp<br>ardlgprlysyiagwgeqvfwagg<br>gwwwvplvapcvgalvgsvvyvl<br>lieahhpeldlhlekadqcqtvdnkl<br>alelegveldlnspkgcpnegqegk<br>kaasgkgeeg
European eel (*Anguilla anguilla*)	Aqp10.2b1	XM_035428387.1	atggactccgtgttcaggagagtccgaatcgagagccttctgctcagacagtgcctggccgagtgtttggg<br>agtctacgtcttgatcgtgtttggctgtggggccgtcgcccaggtgacgacctctgagaacaccaaggggc<br>agtacctgtccatcaacctaggctttgccctaggcaccacctttggagtctacgtctctcgaggcgtgtcag<br>gagctcacctgaaccccgcagtcaccctcagcctgtgcatgctgggaaggcacccttggaggactctgcc	mdsvfrrvriesllllrqclaeclgvyv<br>livfgcgavaqvttsentkgqylsinl<br>gfalgttfgvyvsrgvsgahlnpavtl<br>slcmlgrhpwrtlpfyvffqvfgafl

			cttctatgtcttcttccaggtgtttggggccttccttgccgctgccaccgtcgccttgcagtactacgatgccat<br>tatgcacttcagcaatgggcagctgactgtaacaggaccgactgccaccgctggaattttcgccacttaccc<br>tgcagattacctcagtctgtggagtggctttgtggaccaggtgattgggactggcatgctgctggtgtgcatc<br>ctggctgtgggggaccgcaggaacacccgcatccctcctgagctggcaccgcttctggtgggcctggttg<br>tcctggtgatcgggatatccatgggcgtcaactgtggctacgccctcaacccggccagagacctggggcc<br>ccggctgtacagctatatcgcaggctggggagagcaagtgttctgggcgggggggaggatggtggtgggt<br>ccctctggttgctccctgtgtcggagcgctggtggggtctgtagtgtatgtgctcctgattgaggcacatcac<br>ccagagctggacctccacctggaaaaggctgaccagtgccaaaccgtggacaacaaactggctctagag<br>ctggagggggtcgagctggacctgaattcccccaagggctgccccaacgaagggcaggagggcaaga<br>aggctgcaagtgggaagggtgaggaggggtag	aaatvalqyydaimhfsngqltvtg<br>ptatagifatypadylslwsgfvdqv<br>igtgmllvcilavgdrrntrippelapl<br>lvglvvlvigismgvncgyalnpar<br>dlgprlysyiagwgeqvfwaggg<br>wwwvplvapcvgalvgsvvyvlli<br>eahhpeldlhlekadqcqtvdnklal<br>elegveldlnspkgcpnegqegkka<br>asgkgeeg
	Aqp10.2b2	XM_035430495.1	atggactccgtgttcaggagagtccgaatccagagccgcctgctcagacagtgcctggccgagtgtttgg<br>gagtctacgtcttgatcgtgtttggctgtggggccgctgcccaggtgacaacctctgagaacaccaagggg<br>cagtacctgtccatcaacctaggctttgccctaggcaccacctttggagtctacgtctctcgaggcgtgtcag<br>gagctcacctgaaccctgcagtcaccctcagcctgtgcatgctgggaaggcacccttggaggactctgcc<br>cttctatgtcttcttccaggtgtttggggccttccttgccgctgccaccgtcgccttgcagtactacgacgcca<br>ttatgcacttcagcaatgggcagctgactgtaacaggaccgactgccaccgctgcaattttcgccacttacc<br>ctgcagattacctcagtctgtggagtggctttgtggaccaggtgattgggactggcatgctgctggtgtgcat<br>cctggccgtggaagaccgcaggaacacgcgcatcccagccgagatggcaccgcctctgttcgggctgg<br>ttgtcctggtgatcgggatgtccatgagcgtcaactgcggcggcgccctcaacccggcccgagacctgg<br>ggccccggctgtacagctatatcgcgggctggggagagcaggtgttctgggcagggggaggatggtgg<br>tgggtccctctggttgctccctgtgtcggagcgctggtggggtctgtagtgtatgtgctcctgattgaggcac<br>atcacccagagctggacctccacctggaaaaggctgaccagtgccaaaccgtggacaacaaactggctc<br>tagagctggagggggtcgagctggacctgaattcccccaagggctgccccaacgaagggcaggaggg<br>caagaaggctgcaagtgggaagggtgaggaggggtag	mdsvfrrvriqsrllrqclaeclgvyv<br>livfgcgaaaqvttsentkgqylsinl<br>gfalgttfgvyvsrgvsgahlnpavtl<br>slcmlgrhpwrtlpfyvffqvfgafl<br>aaatvalqyydaimhfsngqltvtg<br>ptataaifatypadylslwsgfvdqvi<br>gtgmllvcilavedrrntripaemap<br>plfglvvlvigmsmsvncggalnp<br>ardlgprlysyiagwgeqvfwagg<br>gwwwvplvapcvgalvgsvvyvl<br>lieahhpeldlhlekadqcqtvdnkl<br>alelegveldlnspkgcpnegqegk<br>kaasgkgeeg
	Aqp10.2b3	XM_035430494.1	atggactccgtgttcaggagagtccgaatccagagccgtctgctcagacagtgcctggccgagtgtttggg<br>agtctacgtcttgatcgtgtttggctgtggggccgctgcccaggtgacaacctctgagaacaccaaggggc<br>agtacctgtccatcaacctaggctttgccctaggcaccacctttggagtctacgtctctcgaggcgtgtcag<br>gagctcacctgaaccctgcagtcaccctcagcctgtgcatgctgggaaggcacccttggaggactctgcc<br>cttctatgtcttcttccaggtgtttggggcctttctcgctgctgccaccgtcgccttgcagtactacgatgccatt<br>atgcacttcagcaatgggcaactgactgtaacaggaccgactgccaccgctgcaattttcgccacttaccct<br>gcagattacctcagtctgtggagtggcttcgttgaccaggtgattgggactggcacgctgctggtgtgcatc<br>ctggccgtggaagaccgcaggaacacgcgcatcccagccgagatggcaccgcctctgttcgggctggtt<br>gtcctggtgatcgggatgtccatgagcgtaaactgcggcggcgccctcaacccggcccgagacctgggg<br>ccccggctgtacagctatatcgcgggctggggagagcaggtgttctgggcggggggaagatggtggtg<br>ggtccctctggttgctccctgtgtcggagcgctggtggggtctgtagtgtatgtgctcctgattgaggcacat<br>cacccagagctggacctccacctggaaaaggccgaccagtgccaaaccgtggacaacaaactggctcta<br>gagctggagggggtcgagctggacctgaattcccccaagggctgccccaacgaagggcaggagggca<br>agaaggctgcaagtgggaagggtgaggaggggtag	mdsvfrrvriqsrllrqclaeclgvyv<br>livfgcgaaaqvttsentkgqylsinl<br>gfalgttfgvyvsrgvsgahlnpavtl<br>slcmlgrhpwrtlpfyvffqvfgafl<br>aaatvalqyydaimhfsngqltvtg<br>ptataaifatypadylslwsgfvdqvi<br>gtgtllvcilavedrrntripaemapp<br>lfglvvlvigmsmsvncggalnpar<br>dlgprlysyiagwgeqvfwaggrw<br>wwvplvapcvgalvgsvvyvllie<br>ahhpeldlhlekadqcqtvdnklale<br>legveldlnspkgcpnegqegkkaa<br>sgkgeeg

Supplementary Table S4. List of plots data of water and solute permeabilities shown in fig. 2A.

	control	AanAqp10.2b1	AanAqp10.2b2	AanAqp10.2b3	SkaAqp10.2b	CcoAqp10.2b	McyAqp10.2b
P_{water} ($\times 10^{-6}$ cm/s, 100 mosM inside osmotic gradient)	1.40	11.23	8.17	5.78	17.68	10.40	10.92
	1.32	12.54	10.61	5.22	15.76	11.15	8.50
	2.00	7.77	10.11	4.85	23.21	17.64	5.60
	0.85	9.69	6.83	3.72	4.77	8.74	5.14
	1.00	7.84	4.33	2.22	6.81	9.60	3.45
	1.06	13.13	7.94	4.48	8.64	13.32	4.73
	0.62	10.23	8.93	4.70	5.00	10.73	1.63
	0.72	4.43	6.36	3.56	7.45	19.95	5.94
	1.07	5.25	5.99	3.39	6.55	3.41	4.46
	1.02	6.13	8.92	2.72	9.62	24.63	2.01
	0.45	4.54	8.02	2.80	15.30	7.27	5.48
	0.97	3.89	3.09	3.74	18.49	12.84	1.37
	0.61			3.79	7.94		1.71
	1.17				12.26		11.28
	0.78						
	1.13						
	1.23						
	0.46						
	1.24						
	0.28						
$P_{glycerol}$ ($\times 10^{-6}$ cm/s, 180 mM outside solute gradient)	0.21	83.93	46.56	51.24	53.71	57.93	19.23
	0.29	57.56	82.72	27.93	16.53	66.56	37.35
	0.13	140.90	65.19	32.14	47.67	97.84	26.16
	0.60	104.29	59.83	13.42	51.06	44.12	21.32
	0.03	156.24	41.06	28.98	56.71	53.62	18.24
	0.25	75.84	32.78	12.94	44.73	102.33	17.88
	0.02	92.27	58.36	21.21	52.78	139.09	40.83
	0.23	101.23	93.74	12.70	52.22	63.96	15.57
	0.07	54.05	56.42	10.62	62.74	80.20	11.94
	0.11	40.59	81.79	15.21	77.92	94.75	40.85
	0.03	51.44	90.92	14.23	48.29		30.11
	0.06	70.41	50.28	26.91	83.76		38.38
	0.13	65.11	41.97	13.70	85.12		23.65
	0.10	49.80	60.65		39.20		56.47
	0.02	80.94			100.52		
	0.04	64.41			94.03		
	0.18						
	0.14						
	0.41						
P_{urea} ($\times 10^{-6}$ cm/s, 180 mM outside solute gradient)	0.04	11.94	85.16	22.61	3.78	10.45	1.18
	0.55	8.07	44.60	16.33	4.28	8.29	2.31
	0.14	12.35	38.78	13.19	2.40	2.38	3.70
	0.02	9.76	96.05	23.46	6.15	11.44	1.56
	0.23	7.79	75.83	23.64	4.10	11.06	1.08
	0.32	7.19	66.83	10.65	2.44	7.08	1.00
	0.02	6.47	47.63	21.01	3.04	10.72	0.68
	0.01	3.51	50.57	15.56	2.58	16.90	2.10
	0.35	5.58	35.54	4.58	1.90	15.20	0.68
	0.18	2.58	55.88	12.21	6.85	15.24	2.74
	0.03	4.50	25.72	10.69	9.37	2.39	3.57
	0.03	2.35	27.08	10.18	7.59		1.84
	0.08	3.62	48.98	7.30			
	0.03		54.69				
	0.21						
	0.26						
	0.16						
$P_{boric\ acid}$ ($\times 10^{-6}$ cm/s, 180 mM outside solute gradient)	0.05	8.53	38.75	31.58	4.58	5.65	3.70
	0.34	7.51	77.54	22.31	3.80	5.22	1.95
	0.43	9.11	51.73	51.54	1.78	6.48	2.28
	0.36	6.02	75.42	21.31	4.71	6.13	0.23
	0.05	1.80	72.80	15.95	2.69	5.76	0.55
	0.08	3.53	56.35	18.62	3.29	13.58	1.86
	0.04	3.05	29.77	13.02	1.99	9.10	0.61

	0.04	3.73	23.86	52.55	4.30	3.18	0.79
	0.12	1.61	77.34	15.88	6.48	15.80	1.70
	0.12	1.89	57.02	11.24	4.39		5.48
	0.45	0.79	54.66	12.47	5.09		2.57
	0.16	1.90	50.05	16.02	2.34		0.21
	0.07	2.48	93.80	12.62			2.09
	0.13		38.21				
	0.48						
	0.04						
	0.00						
	0.15						
	0.40						
	0.01						

Aan, *Anguilla anguilla*; Ska, *Synaphobranchus kaupii*; Cco, *Conger conger*; Mcy, *Megalops cyprinoides*.

Supplementary Table S5. List of plots data of water and solute permeabilities shown in fig. 4.

P_{water} (× 10^{-6} cm/s, 100 mosM inside osmotic gradient)	control	AanAqp10.2b1	AanAqp10.2b1^{Y205G}
	1.40	11.23	4.01
	1.32	12.54	12.51
	2.00	7.77	5.42
	0.85	9.69	12.07
	1.00	7.84	5.31
	1.06	13.13	4.61
	0.62	10.23	4.38
	0.72	4.43	4.85
	1.07	5.25	10.29
	1.02	6.13	6.97
	0.45	4.54	8.41
	0.97	3.89	18.82
			3.81
$P_{glycerol}$ (× 10^{-6} cm/s, 180 mM outside solute gradient)	control	AanAqp10.2b1	AanAqp10.2b1^{Y205G}
	0.21	83.93	88.85
	0.29	57.56	92.73
	0.13	140.90	82.95
	0.60	104.29	25.99
	0.03	156.24	41.92
	0.25	75.84	56.03
	0.02	92.27	68.81
	0.23	101.23	58.80
	0.07	54.05	60.29
	0.11	40.59	30.87
	0.03	51.44	52.51
	0.06	70.41	23.24
		65.11	
		49.80	
		80.94	
		64.41	
P_{urea} (× 10^{-6} cm/s, 180 mM outside solute gradient)	control	AanAqp10.2b1	AanAqp10.2b1^{Y205G}
	0.04	11.94	122.73
	0.55	8.07	97.37
	0.14	12.35	111.64
	0.02	9.76	93.97
	0.23	7.79	50.97
	0.32	7.19	27.98
	0.02	6.47	32.90
	0.01	3.51	34.95
	0.35	5.58	50.28
	0.18	2.58	52.71
	0.03	4.50	56.41
		2.35	34.30
		3.62	
$P_{boric\ acid}$ (× 10^{-6} cm/s, 180 mM outside solute gradient)	control	AanAqp10.2b1	AanAqp10.2b1^{Y205G}
	0.05	8.53	94.48
	0.34	7.51	126.29
	0.43	9.11	87.02
	0.36	6.02	70.90
	0.05	1.80	52.69
	0.08	3.53	43.98
	0.04	3.05	92.31
	0.04	3.73	103.59
	0.12	1.61	101.88
	0.12	1.89	125.72
	0.45	0.79	54.23
		1.90	
		2.48	

Supplementary Table S6. Water and solute permeabilities of Aqp10s in *Xenopus* oocytes.

Protein	P_{water} (× 10^{-6} cm/s, 100 mosM inside osmotic gradient)	$P_{glycerol}$ (× 10^{-6} cm/s, 180 mM outside solute gradient)	P_{urea} (× 10^{-6} cm/s, 180 mM outside solute gradient)	$P_{boric\ acid}$ (× 10^{-6} cm/s, 180 mM outside solute gradient)
Control	0.97 ± 0.38 (20)	0.16 ± 0.14 (19)	0.16 ± 0.15 (17)	0.18 ± 0.16 (20)
AanAqp10.2b1	8.06 ± 3.14 (12)	80.56 ± 31.38 (16)	6.59 ± 3.20 (13)	4.00 ± 2.72 (13)
AanAqp10.2b2	7.44 ± 2.14 (12)	61.59 ± 18.50 (14)	53.81 ± 20.08 (14)	56.95 ± 19.61 (14)
AanAqp10.2b3	3.92 ± 1.00 (13)	21.63 ± 11.13 (13)	14.72 ± 6.08 (13)	22.70 ± 13.55 (13)
SkaAqp10.2b	11.39 ± 5.55 (14)	60.44 ± 21.52 (16)	4.54 ± 2.30 (12)	3.79 ± 1.35 (12)
CcoAqp10.2b	12.47 ± 5.57 (12)	80.04 ± 27.27 (10)	10.10 ± 4.61 (11)	7.88 ± 3.95 (9)
McyAqp10.2b	5.16 ± 3.10 (14)	28.43 ± 12.22 (14)	1.87 ±1.00 (12)	1.85 ± 1.44 (13)
Control	1.04 ± 0.39 (12)	0.17 ± 0.16 (12)	0.17 ± 0.17 (11)	0.19 ± 0.16 (11)
AanAqp10.2b1	8.06 ± 3.14 (12)	80.56 ± 31.38 (16)	6.59 ± 3.20 (13)	4.00 ± 2.72 (13)
AanAqp10.2b1 Y205G	7.81 ± 4.31 (13)	56.92 ± 22.64 (12)	63.85 ± 31.97 (12)	86.64 ± 26.94 (11)

Values are expressed as the mean ± standard deviation. Numbers in parentheses indicate the total number of oocytes tested. Aan, *Anguilla anguilla*; Ska, *Synaphobranchus kaupii*; Cco, *Conger conger*; Mcy, *Megalops cyprinoides*.

Supplementary Table S7. List of plots data of $^{15}N_2$-labeled urea content shown in fig. 3.

3 min	control	AanAqp10.2b1	AanAqp10.2b2	AanAqp10.2b3
	40.56	90.31	317.57	317.94
	9.45	128.26	302.17	428.24
	32.01	107.42	531.23	41.18
		25.31	374.83	234.74
6 min	16.18	193.85	435.64	391.49
	9.20	232.09	273.36	284.52
	12.57	192.12	368.05	318.62
	0.00	170.71	503.87	383.46
		159.14	817.91	232.79
		142.23	664.65	60.25
		145.78		372.16
		41.62		

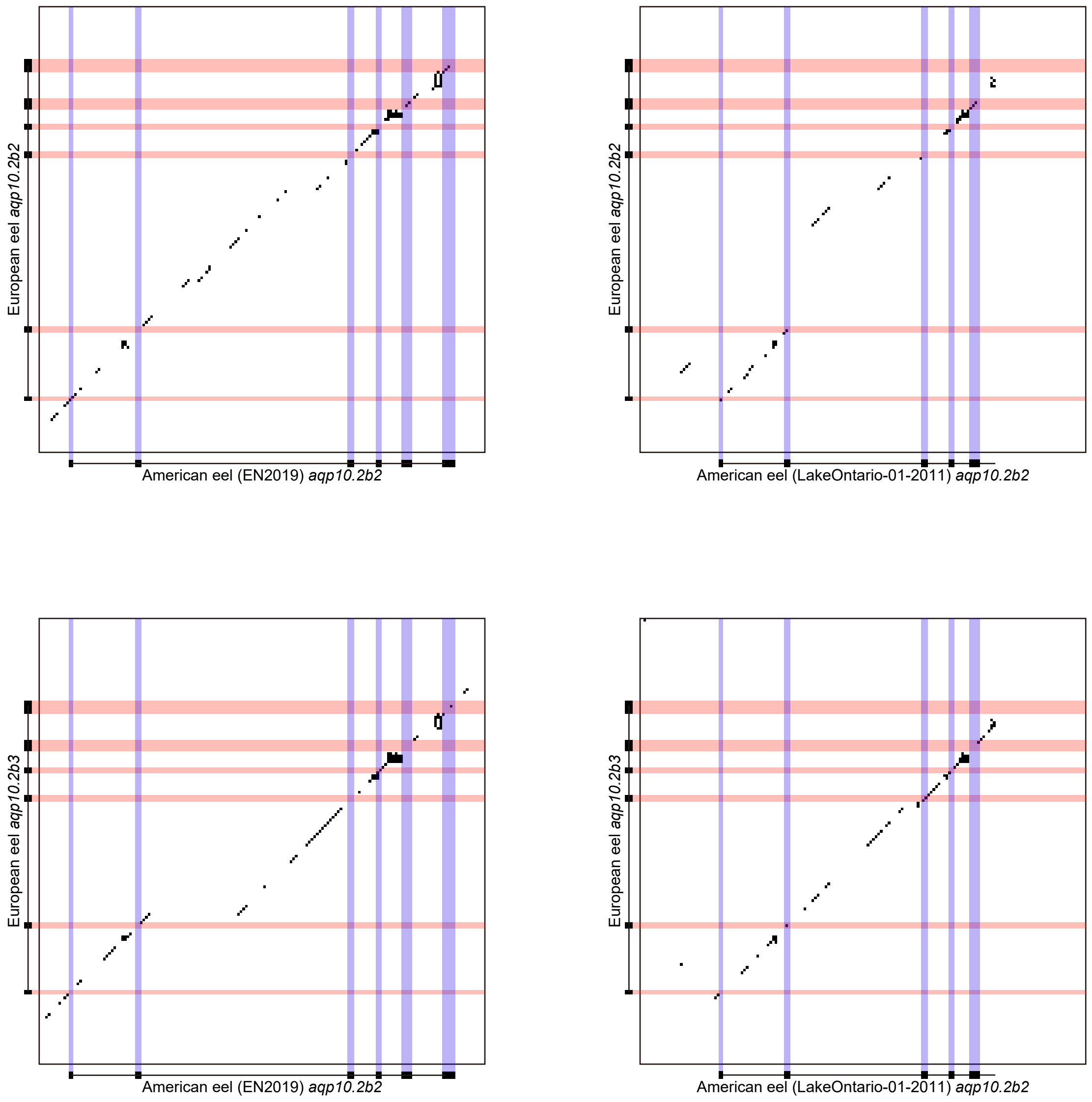

Supplementary Fig. S1. Dot plot analyses of European eel *aqp10.2b2* and *aqp10.2b3* and American eel *aqp10.2b2*. The European eel *aqp10.2b2* and *aqp10.2b3* genes were compared with *aqp10.2b2* from the genome data Anguilla rostrata EN2019 (GCA_018555375.3) (*left*) or that from the genome data Anguilla rostrata LakeOntario-01-2011 (GCA_001606085.1) (*right*). Homologous regions were plotted with dotmatcher program (window size: 20; threshold: 70). The analyzed genomic regions are presented in supplementary table S2.

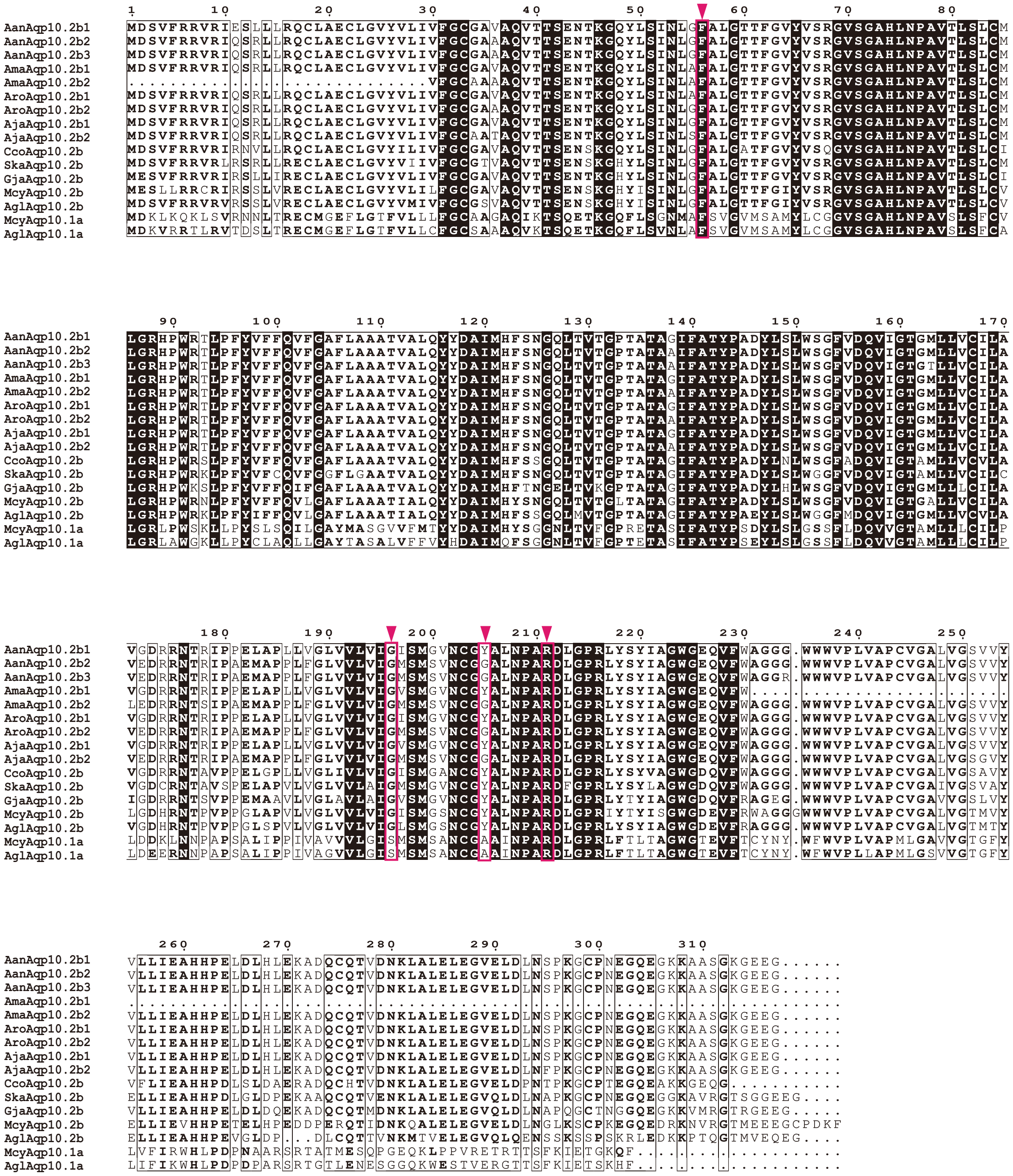

Supplementary Fig. S2. Multiple alignment of the amino acid sequences of Aqp10 in Anguilliformes, roundjaw bonefish, and Indo-pacific tarpon. The four amino acid residues forming the aromatic/arginine (ar/R) selectivity filter are indicated by red arrowheads. Aan, *Anguilla anguilla*; Ama, *Anguilla marmorata*; Aro, *Anguilla rostrata*; Aja, *Anguilla japonica*; Cco, *Conger conger*; Ska, *Synaphobranchus kaupii*; Gja, *Gymnothorax javanicus*; Agl, *Albula glossodonta*; Mcy, *Megalops cyprinoides*.

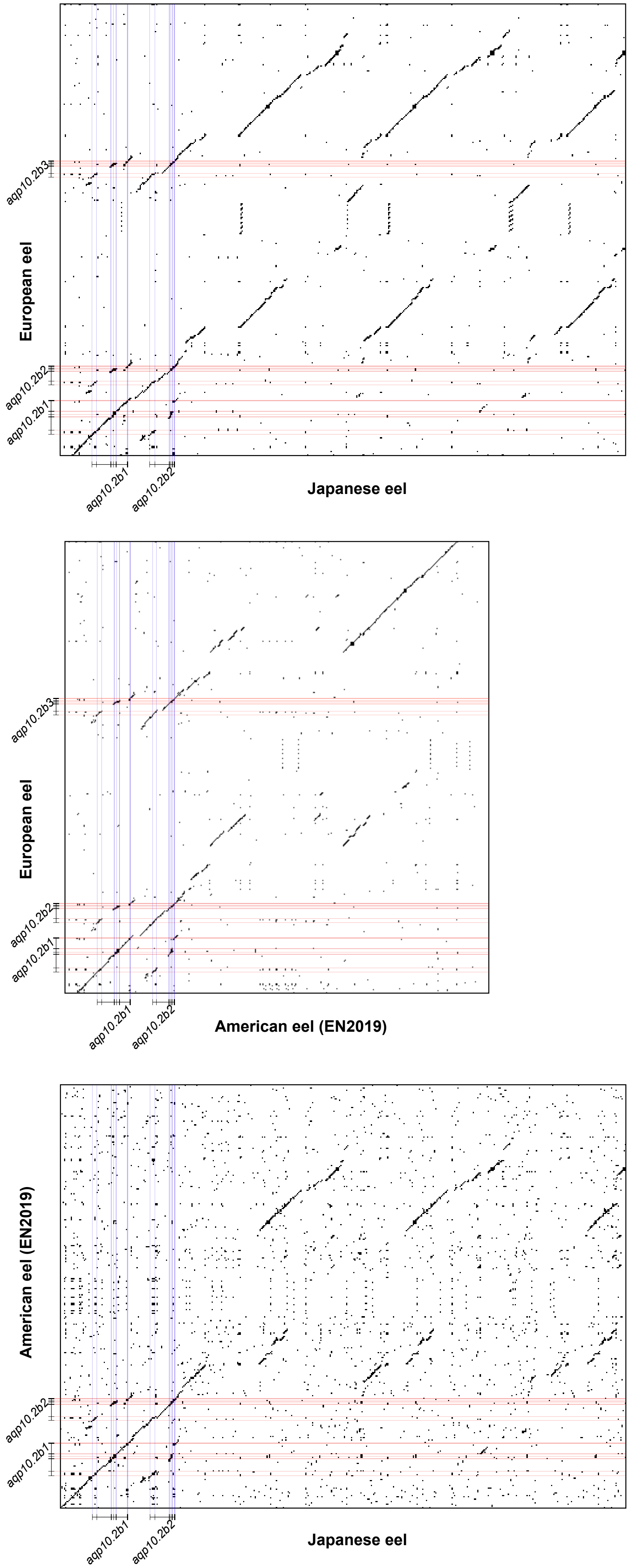

Supplementary Fig. S3. Dot plot analyses of *aqp10.2b* paralogs among European, Japanese, and American eels. Homologous regions were plotted with dotmatcher program (window size: 20; threshold: 70). The analyzed genomic regions are presented in supplementary table S2.

```
AanAqp10.2b1      ATGGACTCCGTGTTCAGGAGAGTCCGAATCGAGAGCCTTCTGCTCAGACA
AanAqp10.2b2      ATGGACTCCGTGTTCAGGAGAGTCCGAATCCAGAGCCGTCTGCTCAGACA
AanAqp10.2b3      ATGGACTCCGTGTTCAGGAGAGTCCGAATCCAGAGCCGTCTGCTCAGACA
CcoAqp10.2b       ATGGATTCCGTGTTCAGGAGAGTTCGGATCAGGAACGTTCTGCTCCGGCA
McyAqp10.2b       ATGGAGTCATTGCTGAGGAGATGCCGCATCAGGAGCAGTTTGGTGAGAGA
McyAqp10.1a       ATGGACAAACTGAAACAGAAGCTGAGTGTGAGGAATAATCTGACCAGGGA

AanAqp10.2b1      GTGCCTGGCCGAGTGTTTGGGAGTCTACGTCTTGATCGTGTTTGGCTGTG
AanAqp10.2b2      GTGCCTGGCCGAGTGTTTGGGAGTCTACGTCTTGATCGTGTTTGGCTGTG
AanAqp10.2b3      GTGCCTGGCCGAGTGTTTGGGAGTCTACGTCTTGATCGTGTTTGGCTGTG
CcoAqp10.2b       GTGCCTGGCGGAGTGTTTGGGAGTCTACATCCTGATCGTGTTTGGCTGTG
McyAqp10.2b       GTGTTTAGCTGAGTGCCTGGGTGTCTATGTCCTGATTCTGTTTGGCTGTG
McyAqp10.1a       GTGTATGGGGGAGTTTCTGGGCACATTTGTTCTGCTGTTGTTTGGTTGTG

AanAqp10.2b1      GGGCCGTCGCCCAGGTGACGACCTCTGAGAACACCAAGGGGCAGTACCTG
AanAqp10.2b2      CGGCCACTGCCCAGGTGACGACCTCTGAGAACACCAAGGGGCAGTACCTG
AanAqp10.2b3      GGGCCGCTGCCCAGGTGACAACCTCTGAGAACACCAAGGGGCAGTACCTG
CcoAqp10.2b       GGGCCGTTGCCCAGGTGACCACCTCTGAGAACAGCAAGGGCCAGTACCTG
McyAqp10.2b       GGGCCGTTGCCCAGGTGACAACGTCTGAGAACAGCAAGGGACACTACATA
McyAqp10.1a       CAGCAGGGGCCCAGATAAAGACCAGTCAAGAGACAAAGGGCCAGTTCCTC

AanAqp10.2b1      TCCATCAACCTAGGCTTTGCCCTAGGCACCACCTTTGGAGTCTACGTCTC
AanAqp10.2b2      TCCATCAACCTAAGCTTTGCCCTAGGCACCACCTTCGGAGTCTACGTCTC
AanAqp10.2b3      TCCATCAACCTAGGCTTTGCCCTAGGCACCACCTTTGGAGTCTACGTCTC
CcoAqp10.2b       TCCATCAACCTGGGCTTCGCCCTGGGGGCCACCTTCGGGGTCTACGTCTC
McyAqp10.2b       TCCATCAACCTGGGCTTTGCGTTGGGCACTACCTTTGGTATCTATGTCTC
McyAqp10.1a       TCCGGCAACATGGCCTTCTCTGTGGGCGTCATGTCTGCCATGTACCTCTG

AanAqp10.2b1      TCGAGGCGTGTCAGGAGCTCACCTGAACCCCGCAGTCACCCTCAGCCTGT
AanAqp10.2b2      TCGAGGCGTGTCAGGAGCTCACCTGAACCCCGCAGTCACCCTCAGCCTGT
AanAqp10.2b3      TCGAGGCGTGTCAGGAGCTCACCTGAACCCTGCAGTCACCCTCAGCCTGT
CcoAqp10.2b       GCAGGGCGTGTCAGGAGCTCATCTGAACCCCGCTGTCACCCTCAGCCTCT
McyAqp10.2b       CAGAGGCGTGTCAGGGGCCCATCTGAACCCGGCAGTGACCCTCAGCCTTT
McyAqp10.1a       TGGGGGTGTCTCTGGGGCTCATCTGAACCCTGCTGTGTCTCTCAGCTTCT

AanAqp10.2b1      GCATGCTGGGAAGGCACCCTTGGAGGACTCTGCCCTTCTATGTCTTCTTC
AanAqp10.2b2      GCATGCTGGGAAGGCACCCTTGGAGGACTCTGCCCTTCTATGTCTTCTTC
AanAqp10.2b3      GCATGCTGGGAAGGCACCCTTGGAGGACTCTGCCCTTCTATGTCTTCTTC
CcoAqp10.2b       GCATCCTGGGAAGGCACCCCTGGAGGAGCCTGCCCTTCTACGTCTTCTTC
McyAqp10.2b       GTGTCCTCGGCAGACACCCCTGGAGAAACCTGCCCTTCTACGTCTTCTTC
McyAqp10.1a       GCGTCTTAGGACGGTTGCCCTGGAGCAAGCTGCTGCCATACTCCCTCTCC

AanAqp10.2b1      CAGGTGTTTGGGGCCTTCCTTGCCGCTGCCACCGTCGCCTTGCAGTACTA
AanAqp10.2b2      CAGGTGTTTGGGGCCTTCCTTGCCGCTGCCACCGTCGCCTTGCAGTACTA
AanAqp10.2b3      CAGGTGTTTGGGGCCTTTCTCGCTGCTGCCACCGTCGCCTTGCAGTACTA
CcoAqp10.2b       CAGGTGTTTGGGGCCTTCCTCGCGGCCGCCACCGTCGCCTTGCAGTACTA
McyAqp10.2b       CAGGTGCTGGGGGCCTTCCTCGCTGCGGCAACCATTGCCCTGCAGTACTA
McyAqp10.1a       CAGATTCTGGGAGCCTACATGGCCTCTGGAGTTGTCTTCATGACATACTA

AanAqp10.2b1      CGATGCCATTATGCACTTCAGCAATGGGCAGCTGACTGTAACAGGACCGA
AanAqp10.2b2      CGATGCCATTATGCACTTCAGCAATGGGCAACTGACTGTAACAGGACCGA
AanAqp10.2b3      CGATGCCATTATGCACTTCAGCAATGGGCAACTGACTGTAACAGGACCGA
CcoAqp10.2b       CGACGCCATCATGCACTTCAGCAGTGGGCAGCTCACTGTGACAGGACCAA
McyAqp10.2b       CGACGCCATAATGCACTATAGCAACGGGCAGTTGACCGTGACTGGACTGA
McyAqp10.1a       TGATGCCATCATGCATTACAGTGGGGGAAACTTGACAGTATTTGGACCCA

AanAqp10.2b1      CTGCCACCGCTGGAATTTTCGCCACTTACCCTGCAGATTACCTCAGTCTG
AanAqp10.2b2      CTGCCACCGCTGCAATTTTCGCCACTTACCCTGCAGATTACCTCAGTCTG
AanAqp10.2b3      CTGCCACCGCTGCAATTTTCGCCACTTACCCTGCAGATTACCTCAGTCTG
CcoAqp10.2b       CAGCGACCGCTGGAATTTTCGCCACTTACCCTGCAGATTACCTGAACCTG
McyAqp10.2b       CCGCCACCGCTGGGATCTTTGCTACTTACCCTGCAGATTACCTGAGCCTG
McyAqp10.1a       GGGAAACTGCCTCCATTTTTGCCACGTACCCATCTGACTATCTGTCTCTT
```

```
AanAqp10.2b1      TGGAGTGGCTTTGTGGACCAGGTGATTGGGACTGGCATGCTGCTGGTGTG
AanAqp10.2b2      TGGAGTGGCTTTGTTGACCAGGTGATTGGGACTGGCATGCTGCTGGTGTG
AanAqp10.2b3      TGGAGTGGCTTCGTTGACCAGGTGATTGGGACTGGCACGCTGCTGGTGTG
CcoAqp10.2b       TGGAGCGGCTTCGCAGACCAGGTCATCGGCACGGCGATGCTGCTGGTGTG
McyAqp10.2b       TGGAGTGGCTTTGTGGACCAGGTGATTGGCACAGGAGCATTGCTGGTGTG
McyAqp10.1a       GGGAGCAGCTTCCTTGACCAGGTTGTCGGCACGGCTATGCTGTTACTCTG

AanAqp10.2b1      CATCCTGGCTGTGGGGGACCGCAGGAACACCCGCATCCCTCCTGAGCTGG
AanAqp10.2b2      CATCCTGGCTGTGGAAGACCGCAGGAACACCCGCATCCCAGCCGAGATGG
AanAqp10.2b3      CATCCTGGCCGTGGAAGACCGCAGGAACACGCGCATCCCAGCCGAGATGG
CcoAqp10.2b       CGTGCTGGCTGTGGGGGACCGCAGGAACACCCCCGTCCCGCCAGAGCTGG
McyAqp10.2b       TATCTTGGCCCTGGGTGACCACAGAAACACCCCTGTCCCACCTGGATTGG
McyAqp10.1a       TATACTGCCCCTGGATGACAAGCTAAACAACCCTGCCCCCTCTGCCTTGA

AanAqp10.2b1      CACCGCTTCTGGTGGGCCTGGTTGTCCTGGTGATCGGGATATCCATGGGC
AanAqp10.2b2      CACCGCCTCTGTTTGGCCTGGTTGTCCTGGTGATCGGAATGTCCATGAGC
AanAqp10.2b3      CACCGCCTCTGTTCGGGCTGGTTGTCCTGGTGATCGGGATGTCCATGAGC
CcoAqp10.2b       GGCCGCTCCTCGTGGGCCTGATCGTCCTGGTGATCGGCATTTCCATGGGC
McyAqp10.2b       CACCGGTTCTAGTAGGCTTGGTGGTCTTGGTGATCGGGATCTCCATGGGC
McyAqp10.1a       TCCCCCCCATTGTGGCAGTGGTGGTCTTGGGAATATCTATGTCCATGTCC

AanAqp10.2b1      GTCAACTGTGGCTACGCCCTCAACCCGGCCAGAGACCTGGGGCCCCGGCT
AanAqp10.2b2      GTCAACTGCGGTGGCGCCCTCAACCCGGCCCGAGACCTGGGGCCCCGGCT
AanAqp10.2b3      GTAAACTGCGGCGGCGCCCTCAACCCGGCCCGAGACCTGGGGCCCCGGCT
CcoAqp10.2b       GCCAACTGCGGCTACGCCCTCAACCCGGCCCGGGACCTGGGCCCCCGCCT
McyAqp10.2b       TCCAACTGTGGATACGCCCTGAACCCAGCCCGAGACCTGGGACCACGGAT
McyAqp10.1a       GCTAACTGTGGGGCGGCCATCAACCCGGCCAGGGACCTGGGGCCCAGACT

AanAqp10.2b1      GTACAGCTATATCGCAGGCTGGGGAGAGCAAGTGTTCTGG---GCGGGGG
AanAqp10.2b2      GTACAGCTATATCGCGGGCTGGGGAGAGCAGGTGTTCTGG---GCGGGGG
AanAqp10.2b3      GTACAGCTATATCGCGGGCTGGGGAGAGCAGGTGTTCTGG---GCGGGGG
CcoAqp10.2b       GTACAGCTACGTCGCGGGCTGGGGAGACCAGGTGTTCTGG---GCGGGCG
McyAqp10.2b       CTACACCTACATCTCAGGCTGGGGTGACGAGGTCTTCAGGTGGGCTGGGG
McyAqp10.1a       GTTCACACTTACTGCAGGCTGGGGGACTGAGGTTTTCACG---TGCTATA

AanAqp10.2b1      GAGGATGGTGGTGGGTCCCTCTGGTTGCTCCCTGTGTCGGAGCGCTGGTG
AanAqp10.2b2      GAGGATGGTGGTGGGTCCCTCTGGTTGCTCCCTGTGTCGGAGCGCTGGTG
AanAqp10.2b3      GAAGATGGTGGTGGGTCCCTCTGGTTGCTCCCTGTGTCGGAGCGCTGGTG
CcoAqp10.2b       GAGGCTGGTGGTGGGTCCCTCTGGTTGCCCCCTGTGTAGGGGCGCTGGTG
McyAqp10.2b       GAGGTTGGTGGTGGGTGCCGCTGGTTGCTCCCTGTGTGGGGGCCCTTGTA
McyAqp10.1a       ACTACTGGTTCTGGGTTCCTTTGGTAGCGCCTATGCTGGGGGCTGTAGTT

AanAqp10.2b1      GGGTCTGTAGTGTATGTGCTCCTGATTGAGGCACATCACCCAGAGCTGGA
AanAqp10.2b2      GGGTCCGTAGTGTATGTGCTCCTGATTGAGGCACATCACCCAGAGCTGGA
AanAqp10.2b3      GGGTCTGTAGTGTATGTGCTCCTGATTGAGGCACATCACCCAGAGCTGGA
CcoAqp10.2b       GGGTCTGCGGTGTACGTGTTCCTAATCGAGGCGCACCACCCGGATCTGTC
McyAqp10.2b       GGGACGATGGTCTATGAGCTTCTCATTGAGGTACACCACCCAGAGACAGA
McyAqp10.1a       GGGACCGGATTCTACCTGGTGTTCATCCGATGGCACCTGCCTGACCCG--

AanAqp10.2b1      CCTCCACCTGGAAAAGGCTGACCAGTGCCAAACCGTGGACAACAAACTGG
AanAqp10.2b2      CCTCCACCTGGAAAAGGCCGACCAGTGCCAAACCGTGGACAACAAACTGG
AanAqp10.2b3      CCTCCACCTGGAAAAGGCCGACCAGTGCCAAACCGTGGACAACAAACTGG
CcoAqp10.2b       CCTCGACGCGGAGCGGGCTGATCAGTGCCACACCGTGGACAACAAGCTGG
McyAqp10.2b       GCTTCACCCGGAAGATGACCCTGAGCGTCAAACCATCGACAACAAGCAGG
McyAqp10.1a       -------------AATGCTGCCAGGAGCAGAACT---GCCACCATGGAGT

AanAqp10.2b1      CTCTAGAGCTGGAGGGGGTCGAGCTGGACCTGAATTCCCCCAAGGGCTGC
AanAqp10.2b2      CTCTAGAGCTGGAGGGGGTCGAGCTGGACCTGAATTCCCCCAAGGGCTGC
AanAqp10.2b3      CTCTAGAGCTGGAGGGGGTCGAGCTGGACCTGAATTCCCCCAAGGGCTGC
CcoAqp10.2b       CCCTGGAGCTGGAGGGGGTAGAACTGGACCCAAATACCCCCAAGGGCTGC
McyAqp10.2b       CCCTGGAGCTGGAGGGGGTGGAACTGGACCTGAATGGCCTCAAGAGCTGC
McyAqp10.1a       CTCAG---CCTGGGGAACAGAAACTG---------CCACCTGTCCGTGAA
```

```
AanAqp10.2b1    CCCAACGAAGGGCAGGAGGGCAAGAAGGCTGCAAGTGGGAAGGGTGAGGA
AanAqp10.2b2    CCCAACGAAGGGCAGGAGGGCAAGAAGGCTGCAAGTGGGAAGGGTGAAGA
AanAqp10.2b3    CCCAACGAAGGGCAGGAGGGCAAGAAGGCTGCAAGTGGGAAGGGTGAGGA
CcoAqp10.2b     CCCACCGAGGGGCAGGAAGCCAAGAAG---------------GGGGAGCA
McyAqp10.2b     CCCAAAGAGGGGCAAGAAGACAGGAAGAATGTGAGGGGGACTATGGAGGA
McyAqp10.1a     ACCAGAACAACCAGCTTTAAAATAGAG------------ACAGGTAAACA

AanAqp10.2b1    GGGGTAG------------------
AanAqp10.2b2    GGGGTAG------------------
AanAqp10.2b3    GGGGTAG------------------
CcoAqp10.2b     GGGGTAG------------------
McyAqp10.2b     GGAGGGCTGTCCAGACAAGTTTTGA
McyAqp10.1a     GTTCTGA------------------
```

Supplementary Fig. S4. List of nucleotide sequences of Aqp10-coding regions used for molecular clock analysis. Aan, *Anguilla anguilla*; Cco, *Conger conger*; Mcy, *Megalops cyprinoides*.

>AanAqp10.2b1
atggactccgtgttcaggagagtccgaatcgagagccttctgctcagacagtgcctggccgagtgtttgggagtctacgtcttgatcgtgtttggctgtggggccgtcgccca
ggtgacgacctctgagaacaccaaggggcagtacctgtccatcaacctaggctttgccctaggcaccacctttggagtctacgtctctcgaggcgtgtcaggagctcacct
gaaccccgcagtcaccctcagcctgtgcatgctgggaaggcacccttggaggactctgcccttctatgtcttcttccaggtgtttggggccttccttgccgctgccaccgtcgc
cttgcagtactacgatgccattatgcacttcagcaatgggcagctgactgtaacaggaccgactgccaccgctggaattttcgccacttaccctgcagattacctcagtctgt
ggagtggctttgtggaccaggtgattgggactggcatgctgctggtgtgcatcctggctgtgggggaccgcaggaacacccgcatccctcctgagctggcaccgcttctgg
tgggcctggttgtcctggtgatcgggatatccatgggcgtcaactgtggctacgccctcaacccggccagagacctggggccccggctgtacagctatatcgcaggctgg
ggagagcaagtgttctgggcgggggaggatggtggtgggtccctctggttgctccctgtgtcggagcgctggtggggtctgtagtgtatgtgctcctgattgaggcacatc
acccagagctggacctccacctggaaaaggctgaccagtgccaaaccgtggacaacaaactggctctagagctggagggggtcgagctggacctgaattcccccaa
gggctgccccaacgaagggcaggagggcaagaaggctgcaagtgggaagggtgaggaggggtag

>AanAqp10.2b2
atggactccgtgttcaggagagtccgaatccagagccgtctgctcagacagtgcctggccgagtgtttgggagtctacgtcttgatcgtgtttggctgtgcggccactgccca
ggtgacgacctctgagaacaccaaggggcagtacctgtccatcaacctaagctttgccctaggcaccaccttcggagtctacgtctctcgaggcgtgtcaggagctcacc
tgaaccccgcagtcaccctcagcctgtgcatgctgggaaggcacccttggaggactctgcccttctatgtcttcttccaggtgtttggggccttccttgccgctgccaccgtcg
ccttgcagtactacgatgccattatgcacttcagcaatgggcaactgactgtaacaggaccgactgccaccgctgcaattttcgccacttaccctgcagattacctcagtctg
tggagtggctttgttgaccaggtgattgggactggcatgctgctggtgtgcatcctggctgtggaagaccgcaggaacacccgcatcccagccgagatggcaccgcctct
gtttggcctggttgtcctggtgatcggaatgtccatgagcgtcaactgcggtggcgccctcaacccggcccgagacctggggccccggctgtacagctatatcgcgggctg
gggagagcaggtgttctgggcgggggaggatggtggtgggtccctctggttgctccctgtgtcggagcgctggtggggtccgtagtgtatgtgctcctgattgaggcacat
cacccagagctggacctccacctggaaaaggccgaccagtgccaaaccgtggacaacaaactggctctagagctggagggggtcgagctggacctgaattcccccca
agggctgccccaacgaagggcaggagggcaagaaggctgcaagtgggaagggtgaagaggggtag

>AanAqp10.2b3
atggactccgtgttcaggagagtccgaatccagagccgtctgctcagacagtgcctggccgagtgtttgggagtctacgtcttgatcgtgtttggctgtggggccgctgccca
ggtgacaacctctgagaacaccaaggggcagtacctgtccatcaacctaggctttgccctaggcaccacctttggagtctacgtctctcgaggcgtgtcaggagctcacct
gaaccctgcagtcaccctcagcctgtgcatgctgggaaggcacccttggaggactctgcccttctatgtcttcttccaggtgtttggggcctttctcgctgctgccaccgtcgcc
ttgcagtactacgatgccattatgcacttcagcaatgggcaactgactgtaacaggaccgactgccaccgctgcaattttcgccacttaccctgcagattacctcagtctgtg
gagtggcttcgttgaccaggtgattgggactggcacgctgctggtgtgcatcctggccgtggaagaccgcaggaacacgcgcatcccagccgagatggcaccgcctct
gttcgggctggttgtcctggtgatcgggatgtccatgagcgtaaactgcggcggcgccctcaacccggcccgagacctggggccccggctgtacagctatatcgcgggct
ggggagagcaggtgttctgggcgggggaagatggtggtgggtccctctggttgctccctgtgtcggagcgctggtggggtctgtagtgtatgtgctcctgattgaggcaca
tcacccagagctggacctccacctggaaaaggccgaccagtgccaaaccgtggacaacaaactggctctagagctggagggggtcgagctggacctgaattccccc
aagggctgccccaacgaagggcaggagggcaagaaggctgcaagtgggaagggtgaggaggggtag

>AmaAqp10.2b1
atggactccgtgttcaggagagtccgaatccagagccgtctgctcaggcagtgcctggccgagtgtttgggagtctacgtcttgattgtgtttggctgtggggccgtcgccca
ggtgacgacctctgagaacaccaaggggcagtacctgtccatcaacctagcctttgccctaggcaccacctttggagtctacgtctctcgaggcgtgtcaggagctcacct
gaaccctgcagtcaccctcagcctgtgcatgctgggaaggcacccttggaggactctgcccttctatgtcttcttccaggtgtttggggccttccttgccgctgccaccgtcgc
cttgcagtactacgatgccattatgcacttcagcaatgggcagctgacagtaacaggaccgactgccaccgctggaattttcgccacttaccctgcagattacctcagtctgt
ggagtggctttgttgaccaggtgattgggactggcatgctgctggtgtgcatcctggctgtgggggaccgcagaaacacccgcatccctcccgagctggcaccgcttctgg
tgggcctggttgtcctggtgatcggggtgtccatgggcgtcaactgtggctacgccctcaacccagcccgagacctggggccccggctgtacagctatatcgcaggctgg
ggagagcaggtgttctgg

>AmaAqp10.2b2
gtgtttggctgtgcggccgccgcccaggtgacgacctctgagaacaccaaggggcagtacctgtccatcaacctagcctttgccctaggcaccaccttcggagtctacgt
ctctcgaggcgtgtcaggagctcacctgaaccctgcagtcaccctcagcctgtgcatgctgggaaggcacccttggaggactctgcccttctatgtcttcttccaggtgtttgg
ggcctttctcgctgctgccactgtcgccttgcagtactatgatgccattatgcacttcagcaatgggcaactgactgtaacaggaccgactgccaccgctgcaattttcgcca
cttaccctgcagattacctcagtctgtggagtggctttgttgaccaggtgattgggactggcatgctgctggtgtgcatcctggctctggaagaccgcaggaacacctccatc
ccagccgagatggcaccgcctctgtttggcctggttgtcctggtgatcgggatgtccatgagcgtcaactgtggcggcgccctcaacccggcccgagacctggggcccc
ggctgtacagctatatcgcaggctggggagagcaagtgttctgggcgggggaggatggtggtgggtccctctggttgctccctgtgtcggagcgctggtggggtccgta
gtgtatgtgctcctgattgaggcacatcacccagagctggacctccacctggaaaaggccgaccagtgccaaaccgtggacaacaaactggctctagagctggaggg
ggtcgagctggacctgaattcccccaagggctgccccaacgaagggcaggagggcaagaaggctgcaagtgggaagggtgaggaggggtag

>AroAqp10.2b1
atggactccgtgttcaggagagtccggatccagagccgtctgctcagacagtgcctggccgagtgtttgggagtctacgtcttgatcgtgtttggctgtggggccgtcgccca
ggtgacgacctctgagaacaccaaggggcagtacctgtccatcaacctagcctttgccctaggcaccacctttggagtctatgtctctcgaggcgtgtcaggagctcacct
gaaccccgcagtcaccctcagcctgtgcatgctgggaaggcacccttggaggactctgcccttctatgtcttcttccaggtgtttggggccttccttgccgctgccaccgtcgc
cttgcagtactacgatgccattatgcacttcagcaatgggcaactgactgtaacaggaccgactgccaccgctggaattttcgccacttaccctgcagattacctcagtctgt
ggagtggctttgtggaccaggtgattgggactggcatgctgctggtgtgcatcctggctgtgggggaccgcaggaacacccgcatccctcctgagctggcaccgcttctgg
tgggcctggttgtcctggtgatcgggatatccatgggcgtcaactgtggctacgccctcaacccggccagagacctggggccccggctgtacagctatatcgcgggctgg
ggagagcaagtgttctgggcgggggaggatggtggtgggtccctctggttgctccctgtgtcggagcgctggtggggtctgtagtgtatgtgctcctgattgaggcacatc
acccagagctggaccttcacctggaaaaggctgaccagtgccaaaccgtggacaacaaactggctctagagctggagggggtcgagctggacctgaattcccccaa
gggctgccccaacgaagggcaggagggcaagaaggctgcaagtgggaagggtgaggaggggtag

>AroAqp10.2b2
atggactccgtgttcaggagagtccggatccagagccgtctgctcagacagtgcctggccgagtgtttgggagtctacgtcttgatcgtgtttggctgtggggccgctgccca
ggtgacaacctctgagaacaccaaggggcagtacctgtccatcaacctaggctttgccctaggcaccacctttggagtctacgtctctcgaggcgtgtcaggagctcacct
gaatcctgcagtcaccctcagcctgtgcatgctgggaaggcacccttggaggactctgcccttctatgtcttcttccaggtgtttggggccttccttgccgctgccactgtcgcct
tgcagtactatgatgccattatgcacttcagcaatgggcaactgactgtaacaggaccgactgccaccgctgcaattttcgccacttaccctgcagattacctcagtctgtgg
agtggcttcgttgaccaggtgattgggactggcatgctgctggtgtgcatcctggccgtggaagaccgcaggaacacccgcatcccagccgagatggcaccgcctctgtt
tggcctggttgtcctggtgatcgggatgtccatgagcgtaaactgcggcggcgccctcaacccggcccgagacctggggccccggctgtacagctatatcgcgggctgg
ggagagcaggtgttctgggcagggggaggatggtggtgggtccctctggttgctccctgtgttggagcgctggtggggtccgtagtgtatgtgctcctgattgaggcacatc

acccagagctggacctccacctggaaaaggccgaccagtgccaaaccgtggacaacaaactggctctagagctggagggggtcgagctggacctgaattcccccaagggctgccccaacgaagggcaggagggcaagaaggctgcaagtgggaagggtgaggaggggtag

>AjaAqp10.2b1
atggactccgtgttcaggagagtccgaatccagagccgtctgctcagacagtgcctggccgagtgtttgggagtctacgtcttgatcgtgtttggctgtggggccgttgcccaggtgacgacgtctgagaacaccaaggggcagtacctgtccatcaacctaggctttgccctaggcaccaccttcggagtctacgtctctcgaggcgtgtcaggagctcacctgaaccccgcagtcaccctcagcctgtgcatgctgggaaggcacccttggaggactctgcccttctatgtcttcttccaggtgtttggggccttccttgccgctgccaccgtagccttgcagtactacgatgccattatgcacttcagcaatgggcagctgacagtaacaggaccgactgccaccgctggaattttcgccacataccctgcagattacctcagtctgtggagtggctttgtggaccaggtgattgggactggtatgctgctggtgtgcatcctggctgtgggggaccgcaggaacacccgcatccctcccgagctggcaccgcttctggtgggcctggttgtcctggtgatcggggtgtccatgggcgtcaactgcggctacgccctcaacccggcccgagacctggggccccggctgtacagctatatcgcgggctggggagagcaggtgttctgggcgggggaggatggtggtgggtccctctggttgctccctgtgtcggagcgctggtggggtccgtagtgtatgtgctcctgattgaggcacatcacccagagctggacctccacctggaaaaggccgaccagtgccaaaccgtggacaacaaactggctctagagctggagggggtcgagctggacctgaattccccaagggctgccccaacgaagggcaggagggcaagaaggctgcaagtgggaagggtgaagaggggtag

>AjaAqp10.2b2
atggactccgtgttcaggagagtccgaatccagagccgtctgctcagacagtgcctggccgagtgtttgggagtctacgtcttgatcgtgtttggctgtgcggccactgcccaggtgacgacctctgagaacaccaaggggcagtacctgtccatcaacctaagctttgccctaggcaccaccttcggagtctacgtctctcgaggcgtgtcaggagctcacctgaaccccgcagtcaccctcagcctgtgcatgctgggaaggcacccttggaggactctgcccttctatgtcttcttccaggtgtttggggccttccttgccgctgccaccgtcgccttgcagtactacgatgccattatgcacttcagcaatgggcaactgactgtaacaggaccgactgccaccgctgcaattttcgccacttaccctgcagattacctcagtctgtggagtggctttgttgaccaggtgattgggactggcatgctgctggtgtgcatcctggctgtggaagaccgcaggaacacccgcatcccagccgagatggcaccgcctctgtttggcctggttgtcctggtgatcggaatgtccatgagcgtcaactgcggcggcgccctcaacccggcccgagacctggggccccggctgtacagctatatcgcgggctggggagagcaggtgttctgggcgggggaggatggtggtgggtccctctggttgctccctgtgtcggagcgctggtggggtccggagtgtatgtgctcctgattgaggcacatcacccagagctggacctccacctggaaaaggccgaccagtgccaaaccgtggacaacaaactggctctagagctggagggggtcgagctggacctgaatttccccaagggctgccccaacgaagggcaggagggcaagaaggctgcaagtgggaagggtgaagaggggtag

>CcoAqp10.2b
Atggattccgtgttcaggagagttcggatcaggaacgttctgctccggcagtgcctggcggagtgtttgggagtctacatcctgatcgtgtttggctgtggggccgttgcccaggtgaccacctctgagaacagcaagggccagtacctgtccatcaacctgggcttcgccctgggggccaccttcggggtctacgtctcgcagggcgtgtcaggagctcatctgaaccccgctgtcaccctcagcctctgcatcctgggaaggcacccctggaggagcctgcccttctacgtcttcttccaggtgtttggggccttcctcgcggccgccaccgtcgccttgcagtactacgacgccatcatgcacttcagcagtgggcagctcactgtgacaggaccaacagcgaccgctggaattttcgccacttaccctgcagattacctgaacctgtggagcggcttcgcagaccaggtcatcggcacggcgatgctgctggtgtgcgtgctggctgtgggggaccgcaggaacacccccgtcccgccagagctggggccgctcctcgtgggcctgatcgtcctggtgatcggcatttccatgggcgccaactgcggctacgccctcaacccggcccgggacctgggcccccgcctgtacagctacgtcgcgggctggggagaccaggtgttctgggcgggcggaggctggtggtgggtccctctggttgccccctgtgtaggggcgctggtggggtctgcggtgtacgtgttcctaatcgaggcgcaccacccggatctgtccctcgacgcggagcgggctgatcagtgccacaccgtggacaacaagctggccctggagctggagggggtagaactggacccaaatacccccaagggctgccccaccgaggggcaggaagccaagaagggggagcaggggtag

>McyAqp10.2b
Atggagtcattgctgaggagatgccgcatcaggagcagtttggtgagagagtgtttagctgagtgcctgggtgtctatgtcctgattctgtttggctgtggggccgttgcccaggtgacaacgtctgagaacagcaagggacactacatatccatcaacctgggctttgcgttgggcactacctttggtatctatgtctccagaggcgtgtcaggggcccatctgaacccggcagtgaccctcagcctttgtgtcctcggcagacacccctggagaaacctgcccttctacgtcttcttccaggtgctgggggccttcctcgctgcggcaaccattgccctgcagtactacgacgccataatgcactatagcaacgggcagttgaccgtgactggactgaccgccaccgctgggatctttgctacttaccctgcagattacctgagcctgtggagtggctttgtggaccaggtgattggcacaggagcattgctggtgtgtatcttggccctgggtgaccacagaaacacccctgtcccacctggattggcaccggttctagtaggcttggtggtcttggtgatcgggatctccatgggctccaactgtggatacgccctgaacccagcccgagacctgggaccacggatctacacctacatctcaggctggggtgacgaggtcttcaggtgggctgggggaggttggtggtgggtgccgctggttgctccctgtgtgggggcccttgtagggacgatggtctatgagcttctcattgaggtacaccacccagagacagagcttcacccggaagatgaccctgagcgtcaaaccatcgacaacaagcaggccctggagctggagggggtggaactggacctgaatggcctcaagagctgccccaaagaggggcaagaagacaggaagaatgtgagggggactatggaggaggagggctgtccagacaagttttga

>McyAqp10.1a
atggacaaactgaaacagaagctgagtgtgaggaataatctgaccagggagtgtatgggggagtttctgggcacatttgttctgctgttgtttggttgtgcagcaggggcccagataaagaccagtcaagagacaaagggccagttcctctccggcaacatggccttctctgtgggcgtcatgtctgccatgtacctctgtgggggtgtctctggggctcatctgaaccctgctgtgtctctcagcttctgcgtcttaggacggttgccctggagcaagctgctgccatactccctctcccagattctgggagcctacatggcctctggagttgtcttcatgacatactatgatgccatcatgcattacagtgggggaaacttgacagtatttggacccagggaaactgcctccatttttgccacgtacccatctgactatctgtctcttgggagcagcttccttgaccaggttgtcggcacggctatgctgttactctgtatactgcccctggatgacaagctaaacaaccctgccccctctgccttgatcccccccattgtggcagtggtggtcttgggaatatctatgtccatgtccgctaactgtggggcggccatcaacccggccagggacctggggcccagactgttcacacttactgcaggctgggggactgaggttttcacgtgctataactactggttctgggttcctttggtagcgcctatgctgggggctgtagttgggaccggattctacctggtgttcatccgatggcacctgcctgacccgaatgctgccaggagcagaactgccaccatggagtctcagcctggggaacagaaactgccacctgtccgtgaaaccagaacaaccagctttaaaatagagacaggtaaacagttctga

>N20
gtgtttggctgtggggccgttgcccaggtgacgacctctgagaacaccaaggggcagtacctgtccatcaacctaggctttgccctaggcaccacctttggagtctacgtctctcgaggcgtgtcaggagctcacctgaaccctgcagtcaccctcagcctgtgcatgctgggaaggcacccttggaggactctgcccttctatgtcttcttccaggtgtttggggccttccttgccgctgccaccgtcgccttgcagtactacgatgccattatgcacttcagcaatgggcagctgactgtaacaggaccgactgccaccgctggaattttcgccacttaccctgcagattacctcagtctgtggagtggctttgttgaccaggtgattgggactggcatgctgctggtgtgcatcctggctgtgggggaccgcaggaacacccgcatcccacccgagctggcaccgcttctggtgggcctggttgtcctggtgatcgggatgtccatgggcgtcaactgcggctacgccctcaacccggcccgagacctggggccccggctgtacagctatatcgcgggctggggagagcaggtgttctgg

Supplementary Fig. S5. List of nucleotide sequences of Aqp10-coding regions used to calculate the substitution rates. N20 is the the estimated ancestral sequence of Aqp10.2b in *Anguilla* species. Aan, *Anguilla anguilla*; Ama, *Anguilla marmorata*; Aro, *Anguilla rostrata*; Aja, *Anguilla japonica*; Cco, *Conger conger*; Mcy, *Megalops cyprinoides*.

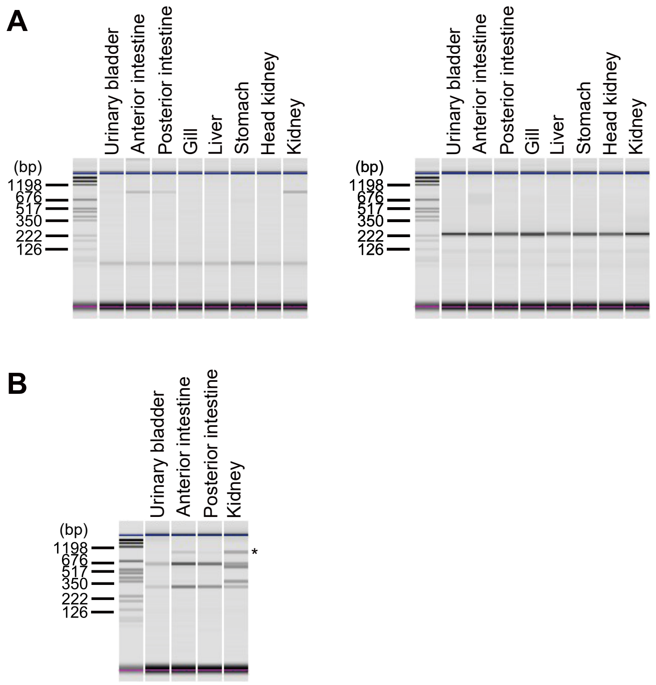

Supplementary Fig. S6. Whole images of a Microchip Electrophoresis system for RT-PCR analysis of Japanese eel *aqp10.2b*. (*A*) Tissue distribution of *aqp10.2b* paralogs in Japanese eel reared in fresh water. PCR products were analyzed by a microchip electrophoresis without restriction enzyme digestion. The generation of pseudo-gel images of the PCR products was facilitated by the microchip electrophoresis system, and the β-actin gene (actb) was utilized as an internal control. (*B*) Expression of *aqp10.2b* paralogs in freshwater-acclimated Japanese eels. PCR products were digested with NcoI and BglI and similarly analyzed by the microchip electrophoresis. An asterisk indicates bands that could not be fully cleaved by the restriction enzyme.
